# A GLUTAMATE CYSTEINE LIGASE Gene *StGSH1* Regulated by StERF10 Enhanced Glutathione Accumulation and Adaptation to Low Phosphorus Stress in Potato

**DOI:** 10.1002/advs.202509143

**Published:** 2025-11-27

**Authors:** Xiaocheng Tian, Mingjun Li, Xiaojia Huang, Markus Wirtz, Xiaohui Zheng, Shilong Fan, Jiang Tian, Shaoqun Liu, Zhonghua Liu, Hongbo Zhao

**Affiliations:** ^1^ Key Laboratory of Biology and Genetic Improvement of Horticultural Crops (South China) Ministry of Agriculture and Rural Affairs College of Horticulture South China Agricultural University Guangzhou 510642 China; ^2^ State Key Laboratory for Crop Stress Resistance and High‐Efficiency Production College of Horticulture Northwest A&F University Yangling Shaanxi 712100 China; ^3^ Centre for Organismal Studies Heidelberg University 69120 Heidelberg Germany; ^4^ Center for Structural Biology School of Life Sciences Tsinghua University Beijing 100084 China; ^5^ Root Biology Center State Key Laboratory for Conservation and Utilization of Subtropical Agro­bioresources College of Natural Resources and Environment South China Agricultural University Guangzhou 510642 China; ^6^ National Research Center of Engineering Technology for Utilization of Functional Ingredients from Botanicals College of Horticulture Hunan Agricultural University Changsha 410128 China

**Keywords:** glutathione, phosphorus, potato, StERF10, *StGSH1*

## Abstract

Potato (*Solanum tuberosum* L.) is a staple global food crop whose growth is constrained by soil phosphorus deficiency. While glutathione (GSH) modulates abiotic stress responses, its precise function and regulation in potato adaptation to low‐phosphorus (LP) remains undefined. This study demonstrated that LP triggers GSH accumulation by up‐regulating GLUTAMATE‐CYSTEINE LIGASE (*StGSH1*), the rate‐limiting enzyme in GSH biosynthesis. Pharmacological inhibition using buthionine sulfoximine (BSO) and exogenous GSH supplementation confirmed the essential role of GSH in LP adaptation. Confocal microscopy showed that StGSH1‐GFP fusions localize to plastids in potato protoplasts. Overexpression and silencing of *StGSH1* demonstrated that it maintained reactive oxygen species (ROS) homeostasis and attenuated LP‐induced damage. *StGSH1* also coordinated membrane lipid remodeling by upregulating both phospholipid catabolic genes (*StNPC/PLD*) and sulfolipid anabolic genes (*StSQD1/2*), shifting lipid flux from phosphatidylethanolamine (PE) to sulfoquinovosyldiacylglycerol (SQDG). Additionally, ETHYLENE RESPONSE FACTOR 10 (StERF10) is identified as a direct transcriptional activator of *StGSH1*. BSO‐mediated GSH depletion reduced the LP adaptation in *StERF10*‐OE plants, whereas GSH supplementation rescued *StERF10*‐RNAi plants, indicating StERF10 conferred LP adaptation largely through *StGSH1*‐mediated GSH biosynthesis. Collectively, these findings establish the StERF10‐StGSH1 module as a critical nexus connecting GSH biosynthesis to LP adaptation, providing a rational target for breeding varieties with enhanced phosphorus use efficiency.

## Graphical abstract

The StERF10‐StGSH1 module coordinates glutathione (GSH) biosynthesis under low‐phosphorus stress. The low‐phosphate‐induced AP2/ERF transcription factor StERF10 directly activates the transcription of *StGSH1* and enhances GSH accumulation. Consequently, GSH scavenges reactive oxygen species and provides sulfur for sulfolipid synthesis, facilitating the substitution of phospholipids and the release of phosphate Pi for plant utilization.

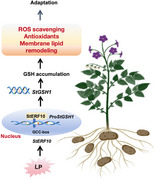

## 1 Introduction

Glutathione (GSH), a sulfur‐containing tripeptide, functions as a central regulator in plant redox homeostasis and stress adaptation.^[^ ^1^, ^2^ ^]^ Within the ascorbate‐GSH (AsA‐GSH) cycle, dehydroascorbate reductase (DHAR) uses GSH to catalyze the reduction of ASA. Concurrently, oxidative reactions drive the conversion of GSH to its disulfide form (GSSG), thereby enabling efficient reactive oxygen species (ROS) scavenging.^[^ ^3^, ^4^ ^]^ The AsA‐GSH cycle facilitates efficient ROS scavenging through two key reactions: dehydroascorbate reductase (DHAR) mediates the GSH‐dependent reduction of dehydroascorbate (DHA) to regenerate ascorbate, while oxidative bursts drive the conversion of GSH to GSSG. GSH effectively mitigates oxidative damage by neutralizing ROS and preventing cellular overoxidation, as demonstrated across plant species under various adversity stresses, such as drought, chilling, salinity, and heavy metals.^[^ ^5^, ^6^, ^7^, ^8^ ^]^ For example, exogenous GSH application significantly mitigates oxidative damage to plant seedlings,^[^ ^9^ ^]^ decreases cold‐induced lipid peroxidation in *Zea mays* and *Eriobotrya japonica*,^[^ ^10^, ^11^ ^]^ and sustains the antioxidant system of *Solanum lycopersicum* under salt stress.^[^ ^12^ ^]^ Beyond its antioxidant functions, GSH enhances stress resilience through species‐specific mechanisms, such as improving drought tolerance in *Arabidopsis* via root architecture modification and stomatal regulation.^[^ ^13^ ^]^ Collectively, these antioxidant and regulatory functions establish GSH as a critical biochemical mediator in plant stress responses.^[^ ^14^ ^]^

The biosynthesis of GSH is regulated by the availability of precursor amino acids (glutamate, cysteine, and glycine) and by the catalytic properties of two ATP‐dependent enzymes: GLUTAMATE‐CYSTEINE LIGASE (GSH1/GCL; EC 6.3.2.2) and GLUTATHIONE SYNTHETASE (GSH2/GS; EC 6.3.2.3). GSH1 catalyzes the ATP‐dependent formation of γ‐glutamylcysteine (γ‐EC) from L‐glutamate and L‐cysteine, marking the initial and rate‐limiting step in GSH biosynthesis.^[^ ^15^, ^16^ ^]^ Notably, GSH1 exerts strict control over cellular GSH levels due to its position as the primary regulatory enzyme in the synthesis pathway.^[^ ^15^, ^17^ ^]^ Consistent with this gatekeeper function, an allelic series of *Arabidopsis GSH1* mutants (*pad2‐1*, *cad2‐1*, and *rax1‐1*) contain only 20%, 30%, and 40% of the wild‐type GSH levels, respectively.^[^ ^18^, ^19^, ^20^ ^]^ Moreover, knockout of *GSH1* in *Arabidopsis* results in embryonic lethality.^[^ ^21^ ^]^ Besides, *GSH1* expression is induced by a variety of abiotic and heavy metal stresses.^[^ ^22^, ^23^ ^]^

As a globally vital staple crop, potato (*Solanum tuberosum* L.) requires substantial phosphorus for growth and tuber initiation, primarily acquired as inorganic phosphate (Pi) from soil.^[^ ^24^, ^25^ ^]^ Paradoxically, although total soil phosphorus is often abundant, its phyto‐availability is limited by low Pi mobility, which markedly constrains productivity.^[^ ^26^ ^]^ LP stress disrupts intracellular homeostasis and elicits excessive ROS accumulation, which in turn inhibits vegetative growth and reduces total dry‐matter production.^[^ ^26^ ^]^ To date, molecular studies on LP tolerance in potato remain scarce.^[^ ^27^, ^28^ ^]^ Although GSH is well established as a key determinant of plant adaptation to abiotic stresses, its specific contribution to LP tolerance has not been investigated. Under Pi starvation, plants rapidly remobilized internal phosphorus reserves. Membrane phospholipids constitute a major internal phosphorus pool. When Pi is limited, phospholipase‐meditated phospholipid hydrolysis, such as phosphatidylethanolamine (PE), releases diacylglycerol (DAG) and free Pi, while sulfoquinovosyldiacylglycerol (SQDG) and digalactosyldiacylglycerol (DGDG) are synthesized to substitute for phospholipids, thereby sustaining membrane integrity and Pi homeostasis.^[^ ^29^ ^]^ Although this Pi starvation‐triggered lipid remodeling has been established, the upstream regulatory networks that orchestrate the switch from phospholipids to sulfo‐/galactolipids remain poorly understood. In particular, whether *GSH1*‐mediated modulation of GSH pools contributes to the remodeling of phospholipid metabolism under LP stress has not yet been addressed.

The APETALA2/Ethylene‐Responsive Factor (AP2/ERF) superfamily transcription factors play widespread roles in plant growth and responses to various abiotic stresses, such as salt, drought, heat, cold, and LP stresses.^[^ ^30^ ^]^ In *Oryza sativa*, *OsERF3* positively regulated drought tolerance,^[^ ^31^ ^]^ whereas *GmERF1* enhanced LP acclimation in *Glycine max* by modulating root architecture and hormone‐mediated phosphate acquisition.^[^ ^32^ ^]^ A genome‐wide survey in *Jatropha curcas* identified 22 *ERF* genes whose expression is rapidly induced by LP stress, indicating the broad involvement of ERFs in plant adaptation to LP stress.^[^ ^33^ ^]^ However, the underlying molecular mechanism through which ERF transcription factors regulate LP adaptation remain elusive.

Despite the established roles of GSH in plant stress response, the molecular mechanisms enabling GSH‐mediated LP adaptation in potato remain unclear. Therefore, this study aims to elucidate the role of potato GSH in LP stress adaptation and the molecular mechanisms regulating its synthesis. We found that GSH accumulation enhanced the adaptation of potato to LP stress, and was driven by rapid transcriptional induction of the glutamate cysteine ligase gene *StGSH1* by LP stress. Further, the enzymatic activity of StGSH1 and its protein crystal structure were characterized. Stable transgenic lines with genetically engineered levels of *StGSH1* demonstrate that *StGSH1‐*dependent GSH accumulation attenuated LP‐induced oxidative damage through ROS homeostasis and promoted membrane lipid remodeling to release available phosphorus, thereby maintaining Pi homeostasis. Furthermore, StERF10, an LP‐induced AP2/ERF transcription factor, was identified as the direct activator of *StGSH1* during LP stress. Consequently, StERF10 was critical for potato adaptation to LP stress. Collectively, this work provides the first mechanistic dissection of the StERF10‐StGSH1‐GSH regulatory cascade that orchestrates cellular redox balance and membrane lipid remodeling to maintain Pi homeostasis during LP stress in potato, and offers a rational target for molecular breeding of phosphorus‐efficient cultivars.

## 2 Results

### 2.1 Glutathione is Essential for Alleviating LP Stress in Potato

**Figure 1:**
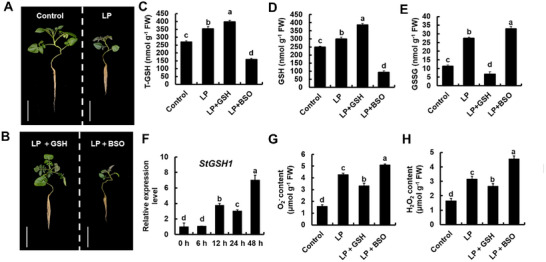
Glutathione (GSH) positively enhances low‐phosphate (LP) adaptation in potato. (A) Phenotypes of wild‐type potato grown under control and LP stress conditions. Plants were grown on Hoagland nutrient solution supplemented with normal P (1.0 mm, KH_2_PO_4_, NP) for 3‐week under short‐day conditions, followed by 15 days of treatment with either 1.0 mm phosphate (Control) or 0.1 mm phosphate (LP) as described in the Experimental Section. Scale bar, 15 cm. (B) Phenotypes of wild‐type potato grown under LP stress condition with the application of 1 mm GSH or GSH inhibition with 0.1 mm BSO. For BSO or GSH treatments, potato plants under LP stress were sprayed with either 1 mm exogenous GSH or 0.1 mm L‐buthionine‐(S,R)‐sulfoximine (BSO), an inhibitor of GCL that suppresses GSH biosynthesis. Foliar sprays were administered every 3 days at 20:00 to avoid GSH photolysis, with phenotypic evaluations conducted 15 days after exposure to LP stress. Scale bar, 15 cm. (C) Total glutathione (T‐GSH), (D) Reduced glutathione (GSH), and (E) Oxidized glutathione (GSSG) content in leaves of potato plants shown in (A,B). (F) The relative transcript steady‐state level of *StGSH1* in leaves of potato subjected to indicated time‐points of LP. (G) O_2_^−^ content, (H) H_2_O_2_ content in leaves of potato shown in (A,B). The bars represent the mean ± SD (*n* = 3 biological replicates; each replicate contained five individual seedlings). One‐way ANOVA test was performed, followed by Tukey's test (*p* < 0.05). Different letters indicate significant difference.

To investigate whether GSH is involved in the response to LP stress (0.1 mm phosphate for 15 days), we examined the GSH content. LP stress significantly promoted the total glutathione (T‐GSH), reduced glutathione (GSH), and oxidized glutathione (GSSG) accumulation, with increases of ≈30%, 22%, and 120% respectively compared to control condition (**Figure** **1**A,C–E). This induction was particularly evident for *StGSH1*, the rate‐limiting enzyme for GSH synthesis, which was significantly induced after 12 h of LP treatment, and accumulated up to seven‐fold higher steady state level at 48 h when compared to the start of the treatment (Figure 1F). Next, to modulate internal GSH steady‐state levels, WT potato plants were treated with foliar applications of either 1 mm exogenous GSH or 0.1 mm L‐buthionine‐(S,R)‐sulfoximine (BSO), an inhibitor of GCL that suppresses GSH biosynthesis. Foliar sprays were administered every 3 days at 20:00 to avoid GSH photolysis, with phenotypic evaluations conducted 15 days after exposure to LP stress (Figure 1B). LP treatment caused significant growth reduction of the wild type (WT) (Figure 1B) that was accompanied by accumulation of O_2_^−^ (Figure 1G) and H_2_O_2_ (Figure 1H). Addition of GSH supported plant growth under LP and caused lowered accumulation of O_2_^−^ and H_2_O_2_ under LP (Figure 1G,H). In contrast, BSO treatments under LP resulted in higher O_2_^−^ and H_2_O_2_ accumulation and decreased plant growth (Figure 1G,H). Exogenous GSH application enhanced reductive buffering capacity under LP stress by directly supplementing reducing equivalents, significantly expanding the T‐GSH and elevating GSH levels (Figure 1C,D). Notably, GSSG content in this treatment was significantly lower than in both LP and control treatment (Figure 1E), likely because exogenous GSH assumed primary antioxidant functions, effectively reducing endogenous GSH consumption. In contrast, BSO treatment led to dramatic collapse of T‐GSH and GSH under LP conditions (Figure 1C,D). This depletion exacerbated oxidative damage, as the burst of ROS accelerated the conversion of GSH to GSSG, leading to explosive accumulation of GSSG (Figure 1C–E). We concluded from these findings that LP drives GSH synthesis through transcriptional activation of *StGSH1*. and that GSH accumulation is essential for plant adaptation to LP stress.

### 2.2 StGSH1 is a Functional GCL Enzyme

**Figure 2:**
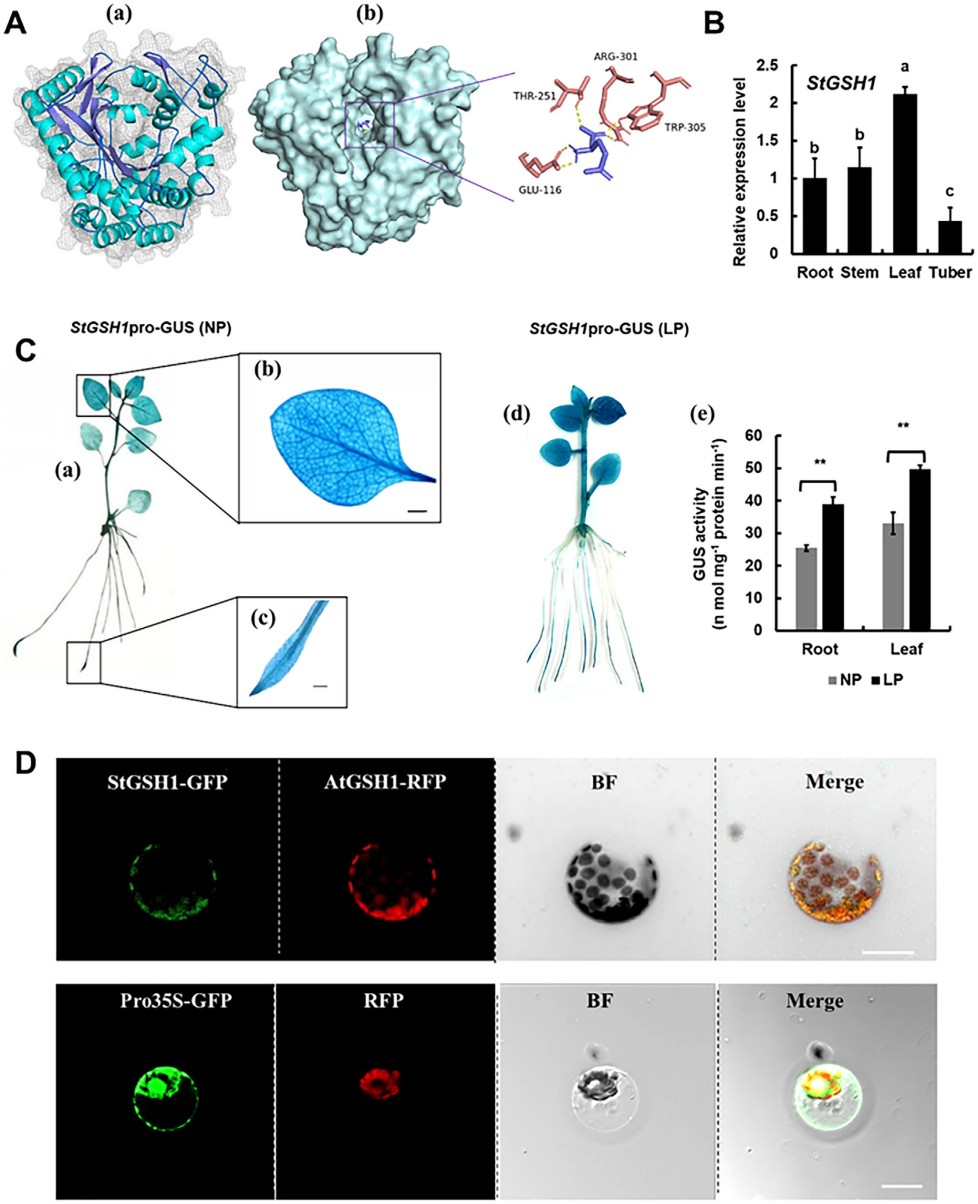
Characterization of StGSH1 structure and expression pattern. (A) Overall crystal structure of StGSH1. α‐Helices are colored Cyan and beta‐strands are colored purple (a). Binding affinities of StGSH1 to L‐glutamate (b). Overall structure with the StGSH1 surface is colored light grey blue on the left. Detailed binding region of L‐glutamate is enlarged on the right, in which the residues of StGSH1 is represented as a light maroon cartoon, and L‐glutamate is indicated as purple sticks. Residues GLU116, THR251, ARG301, and TRP305 are highlighted. Hydrogen bonds are displayed as yellow dotted lines. (B) Relative expression of *StGSH1* in root, stem, leaf, and tuber of potato, which was determined by RT‐qPCR. The expression level of *StGSH1* in root was set to 1.0. The bars represent the mean value ± SD (*n* = 3 biological replicates; each contained 3 individual plants). Statistical tests were performed by one‐way ANOVA, followed by Tukey's test. Different letters indicate significant difference at *p* < 0.05. (C) GUS staining was assessed to confirm active expression of *StGSH1* in potato leaf and root. Histochemical staining for GUS activity in transgenic potato plants expressing GUS under the control of the StGSH1 promoter (*StGSH1*pro‐GUS). Plants were treated for 24 h under control (NP, a–c) and low phosphate (LP, d,e) conditions. (a,d) whole plant, (b) single leaf, and (c) roots. Scale bar, 1 mm. (e) Quantitative analysis of GUS activity in leaves and roots of *StGSH1*pro‐GUS transgenic potato under NP and LP conditions. The bars represent the mean value ± SD (*n* = 3 biological replicates; each contained 5 individual seedlings). Student's *t*‐test was used for statistical significance in (C (e)), **p < 0.01. (D) The subcellular localization of StGSH1 protein in protoplast of potato leaves. The fusion image of the green fluorescence of StGSH1‐GFP and the red fluorescence of the plastid marker AtGSH1‐RFP was shown in merge field. The empty vector (Pro35S‐GFP construct) served as a control. Scale bar, 5 µm.

Because of the remarkable induction of the *StGSH1* gene under LP stress, we characterized the biochemical properties of the StGSH1 protein, which belongs to the GCS2 (Glutamate‐cysteine ligase family 2) superfamily, and shares the highest homology with GSH1 from tomato (Figure S1, Supporting Information). Recombinant StGSH1 protein was expressed in *Escherichia coli* BL21 (Figure S2, Supporting Information) and further purified by ion‐exchange chromatography (Figure S3, Supporting Information) to determine its biochemical properties. The purified StGSH1 protein displayed high GCL activity when cysteine, glutamate, and ATP were used as substrates in the presence of Mg^2+^ (**Table** **1**). As expected, the StGSH1 protein existed as a monomer and homo‐dimer (Figure S3, Supporting Information), with the homo‐dimer being substantially more active than the monomer (Table 1). These biochemical properties align well with the known activation of GSH1s from the Brassicaceae family by oxidation‐induced dimerization.^[^ ^34^ ^]^ The temperature and pH optima of StGSH1 were comparable with the biochemical properties of other plant GCLs, strongly suggesting that StGSH1 is a functional GCL responsible for the bulk of GSH synthesis in potatoes (Figure S4, Supporting Information). To uncover the structure of StGSH1, the purified monomer of StGSH1 was obtained from *Escherichia coli* and refined in a crystal form at a resolution of 2.53 Å (PDB accession number 9 UFC) (**Figure** **2**A). The X­‐ray data collection and structure refinement statistics are shown in Table S1 (Supporting Information). StGSH1 displayed the canonical GCL folding (Figure 2A_a_), which demonstrated functional conservation of the 3D structure of plant GCLs including the predicted GCL dimerization interface and the L‐glutamate binding pocket (Figure S5, Supporting Information; Figure 2A_b_).

**Table 1:** Maximum rate of StGSH1 enzyme reaction (Vmax) and the Km for the substrates.

			*Km*	
	*V*max	L‐Cysteine	L‐Glutamate	ATP
	%		*mm*	
Monomer	61.9 ± 5.8	0.52 ± 0.11	1.46 ± 0.04	2.41 ± 0.36
Dimer	100	0.14 ± 0.02	0.39 ± 0.04	1.00 ± 0.30

Consistent with the vital role of GSH as a redox buffer, the *StSGH1* gene was expressed in all analyzed plant organs and displayed the highest transcript level in leaves, followed by roots, stems, and tubers (Figure 2B). To determine the organ expression pattern of *StGSH1*, a 1.9 kb fragment encompassing the *StGSH1* promoter was fused with the β‐glucuronidase (GUS) reporter and transformed into *Solanum tuberosum* cv. E3 (*StGSH1*pro‐GUS). GUS expression was observed in both roots and leaves in *StGSH1*pro‐GUS transgenic plants (Figure 2C_a–c_). The _Pro_ *StGSH1* was dominantly active in the leaf veins (Figure 2C_b_), and in the crown cells and vascular tissue of roots (Figure 2C_c_). Short‐term LP treatment for 24 h caused substantial accumulation of β‐glucuronidase in leaves and roots of *StGSH1pro‐GUS* transgenic plants, which led to a significant increase in extractable GUS activity compared to normal phosphorus (NP) supply (Figure 2C_d,e_).

In order to investigate the subcellular localization of the StGSH1 protein, StGSH1 was fused to green fluorescent protein (GFP) reporter, and co‐expressed with the plastid marker, AtGSH1‐RFP^[^ ^16^ ^]^ in potato protoplasts. As expected, the StGSH1‐GFP signal overlapped with the red plastid marker signal (Figure 2D), indicating that the StGSH1 protein is localized in the potato plastids. The empty vector carrying 35Spro::GFP served as a control, with its expressed protein localizing to both the cytoplasm and nucleus (Figure 2D). These findings demonstrate that StGSH1 is a plastid‐localized GCL, and the LP‐induced *StGSH1* transcript accumulation may be attributed to the specific transcriptional activation via the here‐identified *StGSH1* promoter.

### 2.3 *StGSH1* Plays a Positive Role in Potato Growth and Adaptation to LP Stress

**Figure 3:**
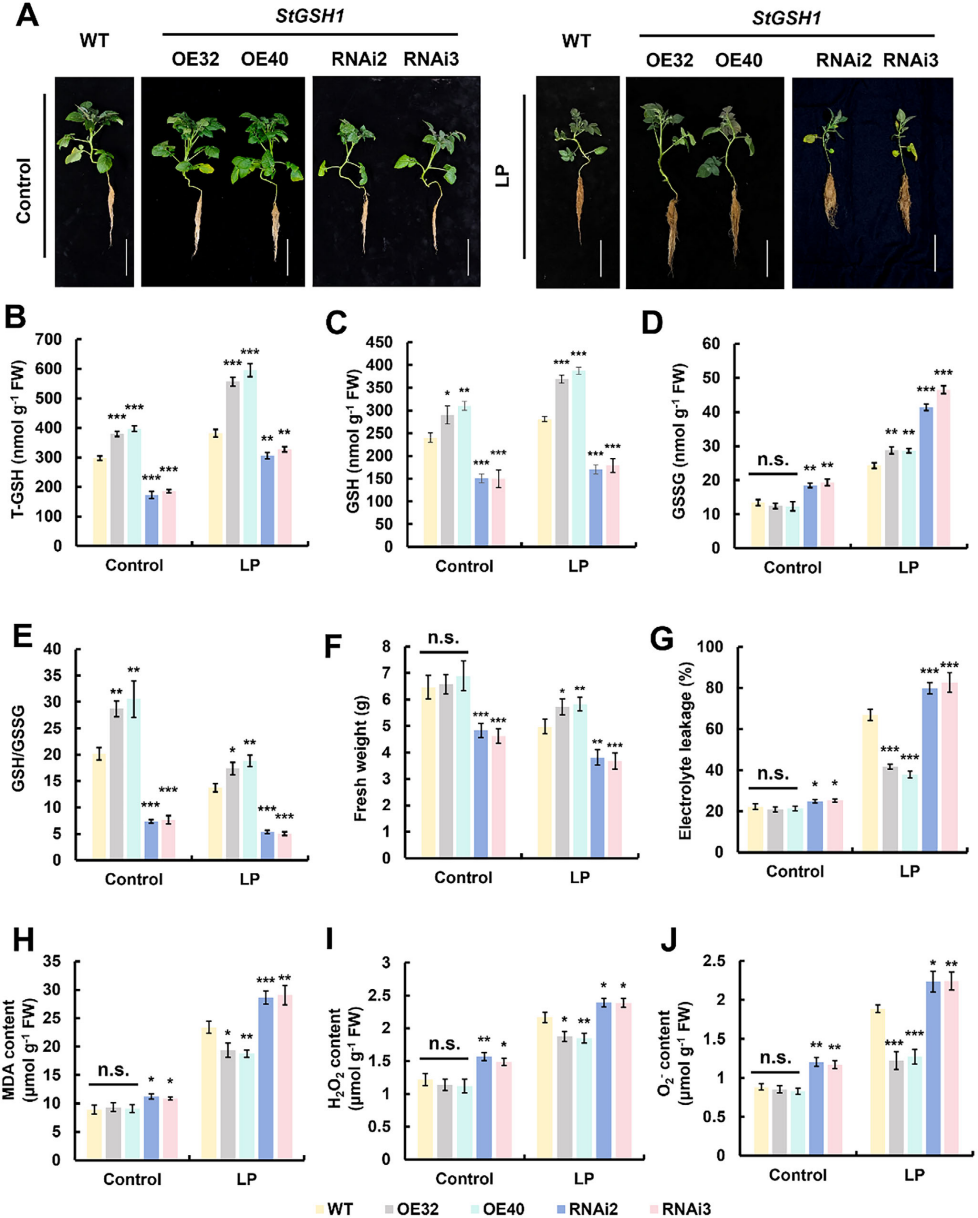
Altering *StGSH1* expression affects plant growth and adaptation to LP stress in potato. (A) Phenotypes of *StGSH1*‐OE (OE32 and OE40), *StGSH1*‐RNAi (RNAi2 and RNAi3), and WT potato plants under LP stress. These plants were grown on Hoagland nutrient solution supplemented with normal phosphate (1.0 mm, KH_2_PO_4_, NP) for 3 weeks, followed by 15 days of normal phosphate (Control) or low phosphate (LP, 0.1 mm KH_2_PO_4_) treatment. Scale bar, 15 cm. (B) Total glutathione (T‐GSH), (C) reduced glutathione (GSH), (D) oxidized glutathione (GSSG), and (E) GSH/GSSG ratio in shoots of wild‐type (WT) and transgenic potato lines (*StGSH1*‐OE: OE32, OE40; *StGSH1*‐RNAi: RNAi2, RNAi3) shown in (A). (F) Fresh weight of shoots of transgenic *StGSH1* potatoes is shown in (A). (G) Electrolyte leakage, (H) MDA content, (I) H_2_O_2_ content, and (J) O_2_^−^ content in shoots of *StGSH1‐*OE and RNAi potatoes shown in (A). The bars represent the mean value ± SD (*n* = 3 biological replicates, each replicate contained 5 individual plants). Statistical tests were performed by Student's *t*‐test (*p < 0.05; **p < 0.01; ***p < 0.001); n.s., not significant.

To provide functional evidence for the role of GSH in the LP stress response, we modulated endogenous GCL activity by genetic engineering of *StGSH1* in potato plants. We identified two potato lines overexpressing *StGSH1* under control of the CaMV35S promoter (OE32 and OE40), and two transgenic lines (RNAi2 and RNAi3) in which the *StGSH1* gene was significantly silenced by specific RNAi constructs introduced via *Agrobacterium*‐mediated transformation (**Figure** **3**A; Figure S6, Supporting Information). Under control conditions, *StGSH1* overexpression significantly expanded both the T‐GSH and GSH pools in shoots, while *StGSH1‐RNAi* lines exhibited reduced T‐GSH and GSH, but increased GSSG relative to WT (Figure 3B,C). Following LP stress, *StGSH1*‐OE lines demonstrated ≈18% higher GSSG content than WT (Figure 3D), indicating that glutathione abundance supports effective antioxidant activity. LP stress reduced the GSH/GSSG ratio in WT, while the *StGSH1*‐OE lines maintained a 26–37% higher ratio than WT (Figure 3E), confirming enhanced redox buffering capacity. In stark contrast, T‐GSH pool in *StGSH1*‐RNAi lines shrank (Figure 3B), and the marked accumulation of GSSG was greater than that of the WT (Figure 3C,D), ultimately triggering a collapse in the GSH/GSSG ratio (Figure 3E). These redox alterations directly governed physiological outcomes: WT plants suffered growth reduction with electrolyte leakage and malondialdehyde (MDA) accumulation (Figure 3G,H), whereas *StGSH1*‐OE attenuated damage, limiting growth inhibition, reducing leakage and MDA content (Figure 3F–H). Conversely, *StGSH1*‐RNAi lines exacerbated stress responses, showing growth suppression (Figure 3F), and increased electrolyte leakage and MDA levels (Figure 3G,H). These findings uncover that the resilience of potatoes toward LP depends on their capability to induce the *StGSH1* gene to cope with LP‐triggered ROS, which are detrimental to membrane lipids.

### 2.4 *StGSH1* Affects ROS Scavenging‐Related Gene Expression and ROS Accumulation under LP Stress

Based on the LP stress‐induced ROS accumulation in potato leaves (Figure 1F,G) and the critical role of GSH as a ROS scavenger in diverse plant species,^[^ ^4^ ^]^ we examined the expression levels of ROS scavenging‐related genes and enzyme activities, including catalase (*CAT*), superoxide dismutase (*SOD*), and ascorbate peroxidase (*APX*) and peroxidase (*POD*), in *StGSH1*‐OE and RNAi potatoes under LP stress. It was found that the *StCAT1*, *StSOD2*, *StPOD66*, and *StAPX1* were significantly induced by LP stress in the WT (Figure S7, Supporting Information). The degree induction of ROS‐scavenging genes positively correlated with the transcript levels of *StGSH1* (Figure S7A–D, Supporting Information). Consistent with gene expression patterns, the enzymatic activities of CAT, SOD, POD, and APX also exhibited significant enhancement (Figure S7E–H, Supporting Information). As expected, the enhanced GSH production in *StGSH1*‐OE lines helped to scavenge the LP stress‐induced accumulation of H_2_O_2_ and O_2_^−^ in leaves (Figure 3I,J), explaining the lowered membrane damage in those lines. The *StGSH1*‐RNAi lines displayed an opposite response compared to *StGSH1*‐OE, which supports the importance of *StGSH1* for scavenging LP‐induced ROS species by GSH (Figure 3; Figure S7, Supporting Information). Furthermore, these results suggest that *StGSH1*‐produced GSH participates in the transcriptional regulation of ROS scavenging‐related genes to maintain redox homeostasis.

### 2.5 *StGSH1* Regulated Phospholipid and Sulfolipid Metabolism in Response to LP Stress

**Figure 4:**
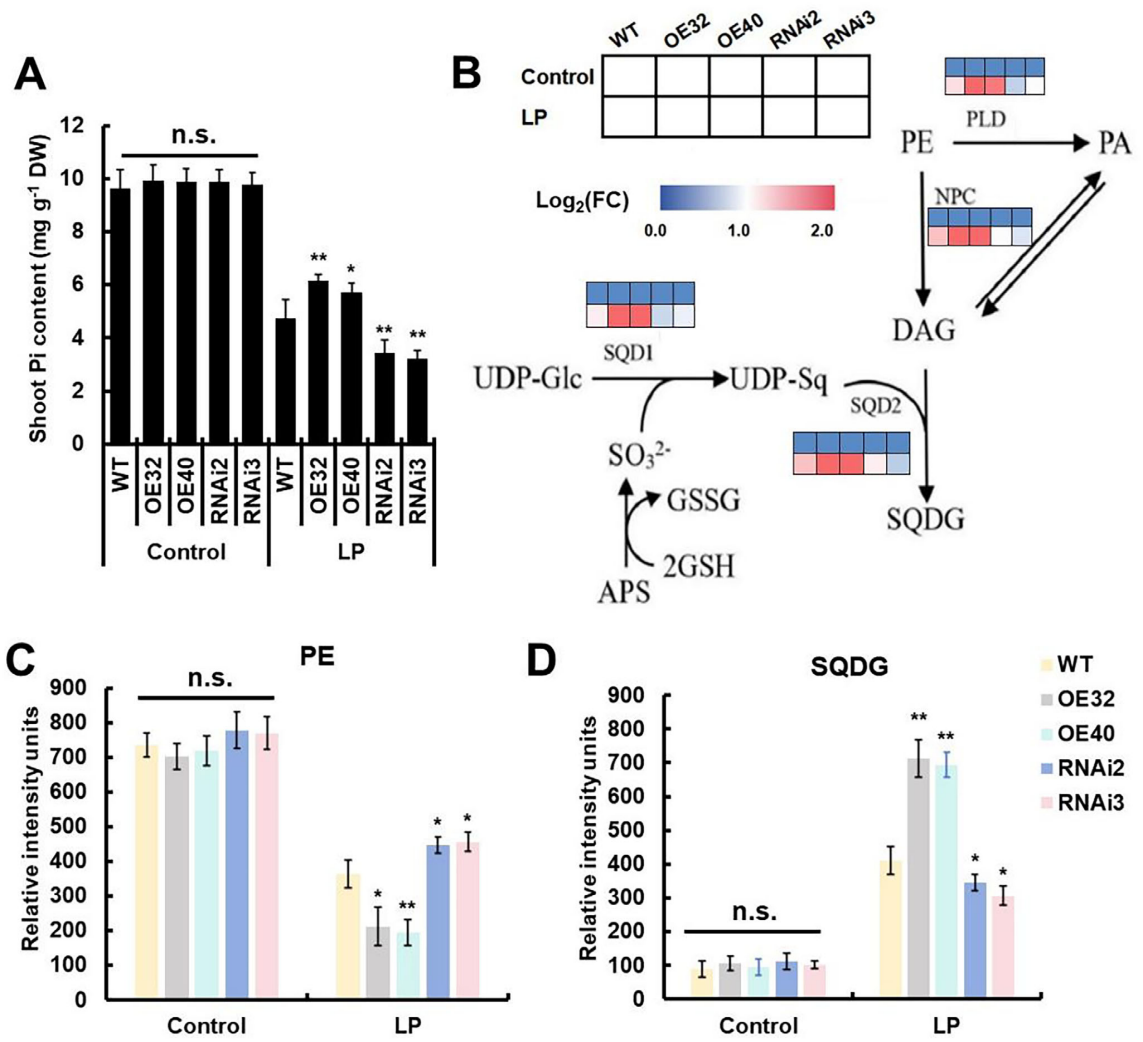
*StGSH1* regulates membrane lipid remodeling in response to LP stress. (A) Pi content in *StGSH1*‐OE and RNAi potato shoots after 15 days of LP stress treatment. *StGSH1*‐OE (OE32 and OE40), *StGSH1*‐RNAi (RNAi2 and RNAi3) and WT potato plants were grown on Hoagland nutrient solution supplemented with normal phosphate (1.0 mm, KH_2_PO_4_, NP) for 3 weeks, followed by 15 days of normal phosphate (Control) or low phosphate (LP; 0.1 mm KH_2_PO_4_) treatment. (B) Relative expression levels of phospholipid degradation genes and sulfolipid synthesis genes in *StGSH1*‐OE and RNAi potato shoots under LP stress were analyzed by RT‐qPCR, with plant growth conditions as described in (A). The color bar represents the gene expression changes (log_2_FC) relative to the wild‐type control under normal phosphorus conditions. FC, fold change. APS, adenosine 5′‐phosphosulphate; DAG, sn‐1,2‐diacylglycerol; GSH, reduced glutathione; GSSG, oxidized glutathione; NPC, non‐specific phospholipase C; PA, phosphatidic acid; PE, phosphatidylethanolamine; PLD, phospholipase D; SQD1, UDP‐sulfoquinovose synthase; SQD2, SQDG synthase; SQDG, sulfolipid sulfoquinovosyldiacylglycerol; UDP‐Glc, uridine diphosphate glucose; UDP‐Sq, uridine diphosphate sulfoquinovose. (C) Total phosphatidylethanolamine (PE) and (D) non‐phosphorus lipid sulfoquinovosyldiacylglycerol (SQDG) content in shoots of *StGSH1*‐RNAi and OE plants with growth conditions as described in (A). Units shown are relative and represent normalized intensities. The bars represent the mean value ± SD (*n* = 3 biological replicates, each replicate contained 5 individual plants). Student's *t*‐test was used to determine statistical significance (*p < 0.05, **p < 0.01); n.s., not significant.

To determine whether GSH is important for coping with LP‐induced ROS formation and its potential impact on Pi uptake, we quantified the contents of Pi in potato leaves with engineered GSH levels under normal and LP conditions. The significantly reduced Pi content in the WT confirmed that the applied LP condition substantially impaired the Pi uptake in potato (**Figure** **4**A). Under control conditions, all lines with engineered GSH metabolism displayed Pi levels similar to the WT. However, under LP stress, the *StGSH1*‐OE lines accumulated significantly more Pi than the WT. Downregulation of *StGSH1* caused the opposite effect on the Pi level, demonstrating that the endogenous Pi level correlates with the ability of potato plants to synthesize GSH via *StGSH1*, specifically when external Pi is limiting (Figure 4A). To exclude potential confounding factors, we quantified total phosphorus (P) (Figure S8A, Supporting Information) and the expression of phosphate starvation‐induced (PSI) genes (Figure S8B–D, Supporting Information). Although LP stress significantly reduced total P content across all genotypes, no significant differences were observed among WT, *StGSH1*‐OE, and *StGSH1*‐RNAi plants (Figure S8A, Supporting Information). Representative PSI genes (*StPHT1;1*, *StPHO1*, *StPAP10*) showed strong upregulation in roots under LP conditions, but their induction levels were comparable across all lines (Figure S8B–D, Supporting Information). These results demonstrate that *StGSH1*‐mediated LP tolerance enhancement does not involve altered total P acquisition.

Phosphorus deficiency triggers a substantial reorganization of membrane lipids, releasing Pi from phospholipids (such as phosphatidylethanolamine, PE) and replacing them with sulfolipids to maintain membrane integrity. For this purpose, PE is converted by phospholipase D (PLD) and non‐specific phospholipase C (NPC) to DAG, which serves as the precursor for sulfolipid biosynthesis by SQDG synthase (SQD2). The sulfur‐containing headgroup for sulfolipid synthesis is provided by uridine diphosphate sulfoquinovose (UDP‐Sq), whose synthesis is catalyzed by SQD1 and requires sulfite.^[^ ^35^, ^36^ ^]^ As expected, LP stress triggered strong transcriptional activation of genes in this conversion pathway, including phospholipid degradation‐related genes *StPLD/StNPC* and sulfolipid synthesis‐related genes *StSQD1/2* (Figure 4B). However, overexpression of *StGSH1* caused a significantly higher induction of these genes by LP in *StGSH1*‐OE compared to WT. Conversely, downregulation of *StGSH1* in RNAi mutants led to a reduced induction of these genes under LP stress (Figure 4B). In addition, the conversion of the phospholipid PE to the sulfolipid SQDG was significantly more efficient in *StGSH1*‐OE shoots when compared to WT, with a 42–47% reduction in PE and a 69–74% increase in SQDG (Figure 4C,D), which explains the higher Pi contents of these plants specifically under LP (Figure 4A). Remarkably, downregulation of *StGSH1* caused lowered PE to SQDG conversion (Figure 4C,D), suggesting that the lower GSH steady‐state levels may have limited the provision of sulfite for UDP‐Sq synthesis, a step that is critical for SQDG production.

### 2.6 The LP‐Induced Tanscription Factor StERF10 Binds to the *StGSH1* Promoter and Activates its Transcription

**Figure 5:**
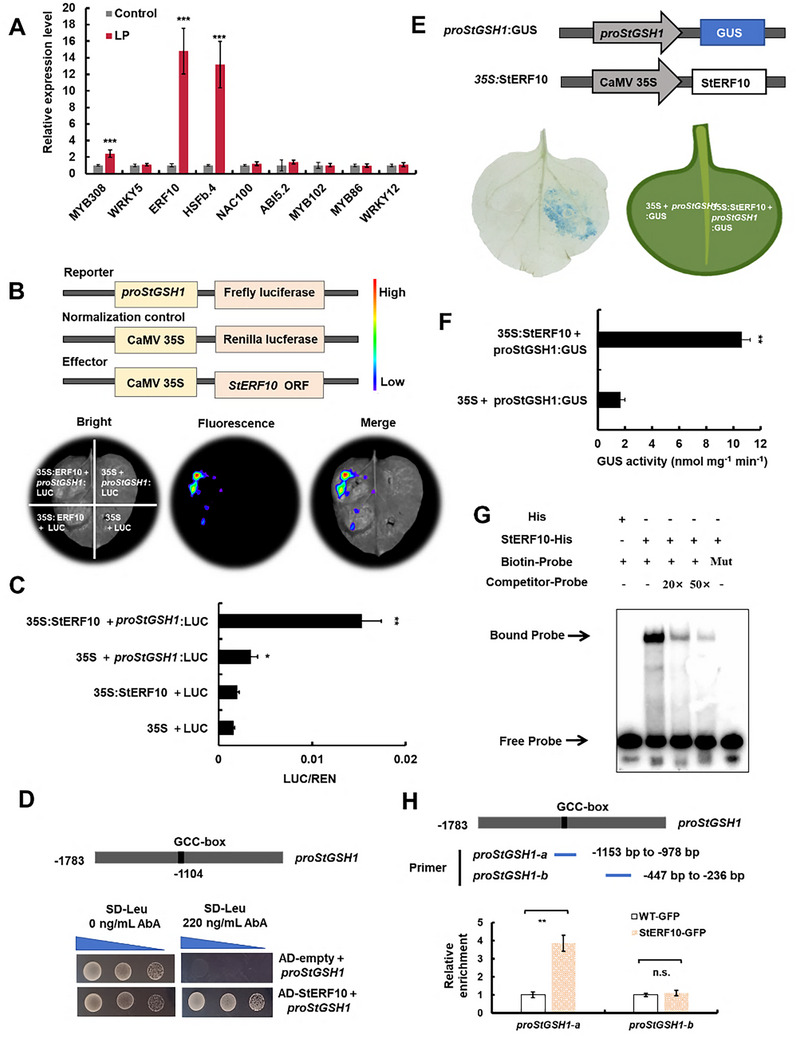
Transcription factor screening and validation of transcriptional regulation of *StGSH1* by StERF10. (A) The relative expression levels of nine candidate TFs including *MYB308, WRKY5, ERF10, HSFb.4, NAC100, ABI5.2, MYB102, MYB86* and *WRKY12* in potato leaves under LP stress. The relative expressions under control were set to 1.0. (B) Dual‐luciferase (LUC) assay showed that *StERF10* could activate *StGSH1* transcription. Schematic diagrams of the effector and reporter constructs are shown above. The *proStGSH1*: LUC or pGreenII 0800‐LUC (empty vector; LUC) was co‐expressed with 35S::StERF10 or 35S (empty vector; pGreenII 62‐SK) in tobacco (*Nicotiana benthamiana*) leaves, respectively. (C) Determination of LUC/REN values of co‐transformed tobacco leaves. Data are from at least three biological replicates. (D) Y1H assay showed that *StERF10* bound to “GCC‐box” element in the *StGSH1* promoter. Empty vector pGADT7 (AD) co‐transformed with the *proStGSH1* in yeast was used as a negative control. Yeast co‐transformed with the *proStGSH1* (pAbAi) and the AD‐StERF10 transformants grew well on SD‐Leu medium supplemented with a selective concentration of 220 ng mL^−1^ Aureobasidin A (AbA). (E‐F) GUS activity assays were performed with tobacco leaves validating that StERF10 activates *StGSH1* transcription in vivo. (G) Electrophoretic mobility shift assay (EMSA) demonstrating StERF10 binding specificity to the GCC‐box element in *StGSH1* promoter. Biotin‐Probe, 5′‐biotin‐labeled probe; “+”, probe/protein present; “‐”, protein omitted; His, His‐tagged recombinant protein (pET‐32a vector); Competitor probe, biotin unlabeled probe; Mut, mutated *StGSH1*‐Pro with 5′‐GCCGCC‐3′ motif substituted by 5′‐aCCaCC‐3′. (H) Chromatin immunoprecipitation quantitative PCR (ChIP‐qPCR) assay was performed to measure the enrichment of StERF10‐GFP on the *StGSH1* promoter using an anti‐GFP antibody. The empty vector (WT‐GFP) served as the control. The eluted DNA fragments were used for amplification and quantification of the promoter sequences of *StGSH1*‐a (*Pro*StGSH1‐a: −1153 to −978 bp) and *StGSH1*‐b (*Pro*StGSH1‐b: −447 to −236 bp). The bars represent the mean value ± SD (*n* = 3 biological replicates). Student's *t*‐test was used to determine statistical significance (*p < 0.05, **p < 0.01, ***p < 0.001); n.s., not significant.

To explore the regulatory molecular mechanism underlying the LP‐induced upregulation of *StGSH1*, Yeast one‐hybrid cDNA library was generated from mRNA isolated from potato leaves. After screening with the full‐length *StGSH1* promoter (pro*StGSH1*) integrated into the yeast genome as bait, 9 candidate transcription factors were identified that potentially bind directly to the *StGSH1* promoter (Table S3, Supporting Information). Out of these candidates, three transcription factors were significantly induced by LP according to RT‐qPCR, including *StMYB308*, *StHSFb.4*, and *StERF10* (**Figure** **5**A).

Next, we applied the dual‐luciferase (LUC) assay using the full‐length *proStGSH1* fused to firefly luciferase as bait to determine whether the three LP‐induced transcription factors could trigger transcription of *StGSH1* in transiently transformed tobacco. After co‐transformation of the reporter construct, *proStGSH1*:LUC, and the effector construct, driving the transcription factor under the control of the 35S promoter, only Ethylene Response Factor 10 (StERF10) was able to induce expression of the firefly luciferase (Figure 5B,C; Figure S9, Supporting Information). Motivated by this finding, we confirmed the direct interaction between StERF10 and *proStGSH1* using a yeast one‐hybrid assay, where *StERF10* was cloned and introduced into yeast containing the pro*StGSH1*‐pAbAi bait. Indeed, the growth of these yeast cells on a selective medium confirmed the direct binding of StERF10 to *proGSH1*, which contains a canonical ERF family transcription factor binding site (GCC‐box)^[^ ^31^ ^]^ located 1104 bp upstream of the *StGSH1* translation start site (ATG) (Figure 5D). Finally, we provided independent evidence for the transcriptional induction of *StGSH1* by StERF10 in vivo by fusing the *proStGSH1* to the GUS reporter. Co‐transformation of the *proStGSH1*:GUS reporter with the *35S::StERF10* effector resulted in a significant increase in the GUS activity when compared to co‐transformation of the *proStGSH1*:GUS reporter with the empty *35S*‐vector control into tobacco (Figure 5E). Additionally, we performed electrophoretic mobility shift assays (EMSA), confirming that the recombinant purified StERF10 protein binds in vitro to DNA probes containing the unique GCC‐box cis‐element within the *StGSH1* promoter (Figure 5G). Concurrently, chromatin immunoprecipitation (ChIP) with anti‐GFP antibody followed by qPCR was conducted on StERF10‐GFP‐expressing potato leaves. The ChIP‐qPCR assay revealed significant enrichment of the pro*StGSH1‐a* fragment (containing the GCC‐box binding site) in immunoprecipitated chromatin from StERF10‐GFP leaves, whereas no enrichment was observed for the pro*StGSH1‐b* fragment (Figure 5H). In summary, these results provide compelling evidence demonstrating a novel regulatory mechanism whereby the LP‐induced transcription factor StERF10 functions as a direct transcriptional activator by binding to the GCC‐box in the promoter to regulate *StGSH1* expression.

### 2.7 *StERF10* Regulated GSH Content and Adaptation to LP Stress in Potato

**Figure 6:**
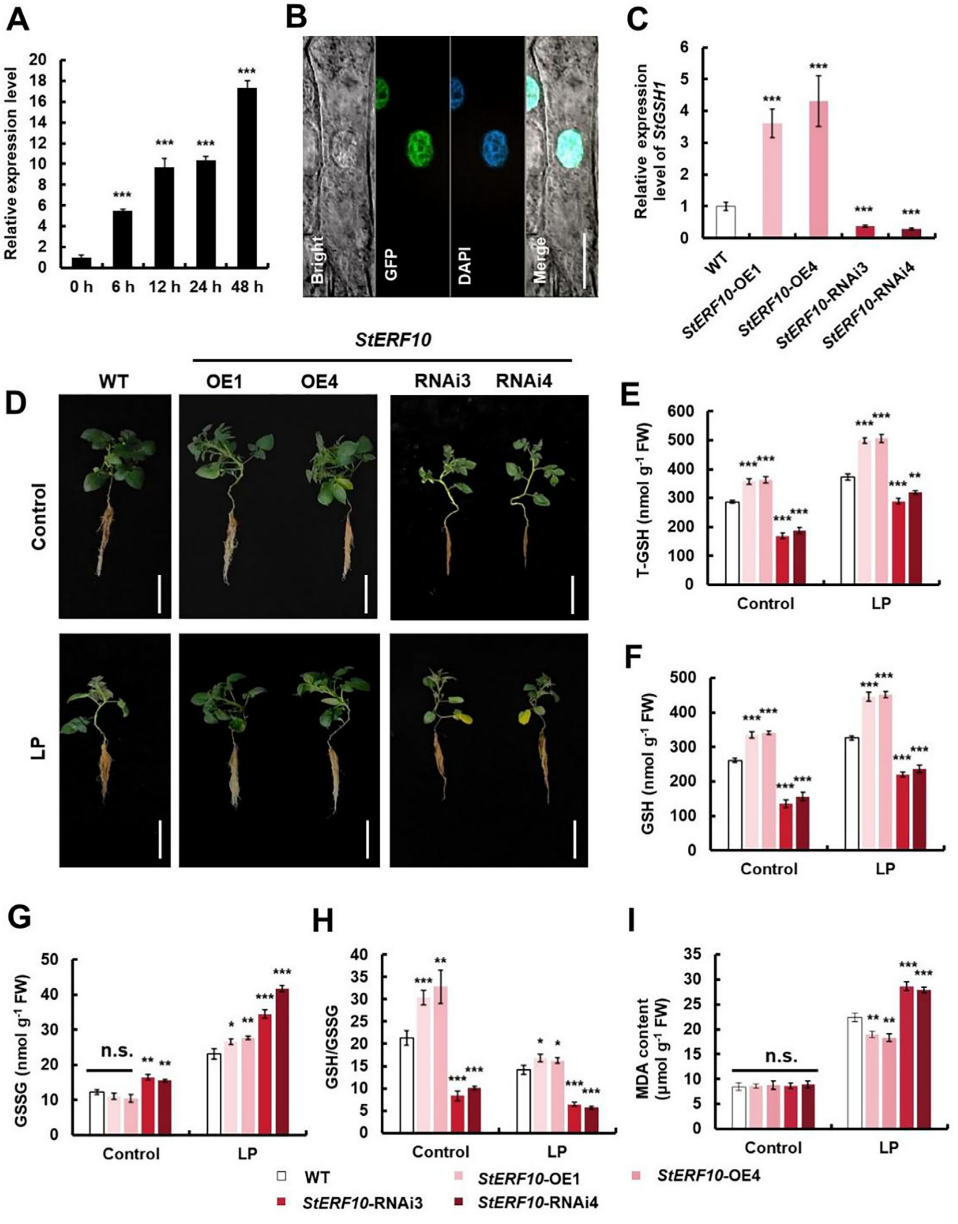
*StERF10* affects GSH content and regulates adaptation to LP stress in potato. (A) The relative expression of *StERF10* in leaves of WT potatoes subjected to indicated time‐points of LP. Plants were pre‐incubated under normal Pi (1 mm) for 3 weeks. The expression at 0 h was set to 1.0. (B) Subcellular localization of StERF10 protein was analyzed in roots of 1‐week‐old tissue‐cultured potato seedlings stably expressing 35S::StERF10::GFP fusion construct using confocal microscopy (excitation 488 nm). The colocalization of the StERF10::GFP fluorescence (green) and the DAPI fluorescence (blue) signals shows that the fusion protein is exclusively localized to the nucleus under control conditions, as expected for a transcription factor. Scale bar, 5 µm. (C) The relative expression of *StGSH1* in leaves of overexpressed (OE1 and OE4) and silenced (RNAi3 and RNAi4) *StERF10* transgenic potatoes under normal Pi treatment conditions by RT‐qPCR analysis. (D) Phenotypes of WT, *StERF10*‐OE (OE1 and OE4) and *StERF10*‐RNAi (RNAi3 and RNAi4) transgenic potatoes under control and LP treatment conditions. These plants were grown on Hoagland nutrient solution supplemented with normal phosphate (1.0 mm, KH_2_PO_4_, NP) for 3 weeks, followed by 15 days of normal phosphate (Control) or low phosphate (LP; 0.1 mm KH_2_PO_4_) treatment. Scale bar, 15 cm. (E) Total glutathione (T‐GSH), (F) reduced glutathione (GSH), (G) oxidized glutathione (GSSG), and (H) GSH/GSSG ratio in shoots of WT, *StERF10*‐OE and *StERF10*‐RNAi plants shown in (D). (I) Malondialdehyde (MDA) content in shoots of WT, *StERF10*‐OE and *StERF10*‐RNAi potato is shown in (D). The bars represent the mean value ± SD (*n* = 3 biological replicates, each replicate contained five individual plants). Student's *t*‐test was used to determine statistical significance (*p < 0.05, **p < 0.01, ***p < 0.001); n.s., not significant.

To provide direct evidence that *StERF10*, an ERF belonging to the AP2 superfamily (Figure S10, Supporting Information), controls the transcription of *StGSH1* in plants upon LP stress, we tested if *StERF10* and *StGSH1* are expressed in the same organs. The *StERF10* transcript was detectable in all analyzed plant organs by RT‐qPCR and displayed a similar expression pattern to *StGSH1* (Figure S11, Supporting Information; Figure 2B). A time course analysis uncovered that *StERF10* is rapidly induced in leaves of 3‐week‐old hydroponically grown plants subjected to LP stress (**Figure** **6**A). The transcriptional induction of *StERF10* occurred before the accumulation of *StGSH1* transcript (Figure 1F), strongly suggesting that the direct binding of StERF10 to the *proStGSH1* stimulates *StGSH1* transcription upon LP stress (Figure 5). Next, we tested the subcellular localization of StERF10 fused to GFP in transgenic potato stably expressing 35S::StERF10::GFP, and found that StERF10‐GFP was localized in the nucleus (Figure 6B). To examine whether altered *StERF10* expression affects adaptation to LP stress in potato, we generated two *StERF10* overexpression lines (*StERF10*‐OE1/4) and two *StERF10* silenced lines (*StERF10*‐RNAi3/4) (Figure 6D), in which the expected alterations of *StERF10* transcript levels were confirmed by RT‐qPCR (Figure S14, Supporting Information). The expression level of *StGSH1* was noticeably up‐regulated in *StERF10*‐OE lines, but lowered in *StERF10*‐RNAi lines compared with WT under regular Pi supply (Figure 6C). Consistent with these findings, T‐GSH and GSH levels increased significantly in shoots of *StERF10*‐OE lines, while *StERF10*‐RNAi lines displayed significantly lower T‐GSH and GSH, but higher GSSG levels when compared to WT (Figure 6E–G). Concurrently, the *StERF10*‐RNAi lines displayed significant decreases in fresh weight of both shoots and roots (Figure S13, Supporting Information). To further determine the effect of *StERF10* on LP stress adaptation, we subjected the WT, *StERF10*‐OE, and RNAi lines to LP stress for 15 days (Figure 6D). As expected, LP stress caused T‐GSH, GSH, and GSSG accumulation in the WT. This LP‐induced GSH accumulation was reinforced in the *StERF10*‐OE lines, with a significantly higher GSH/GSSG ratio than WT reflecting a stronger reductive state, but was diminished in the *StERF10*‐RNAi lines (Figure 6E–H). Consistent with the improved redox state, *StERF10*‐OE lines exhibited stronger adaptation to LP stress compared to the WT, which correlated with lower MDA accumulation in the *StERF10*‐OE lines when compared to WT. In contrast, the opposite effects were observed in *StERF10*‐RNAi lines (Figure 6I). These findings demonstrate that *StERF10* induces *StGSH1* transcription, promotes GSH accumulation, and enhances potato adaptation to LP stress.

### 2.8 *StERF10* Enhanced Potato Adaptation to LP Stress in a GSH‐Dependent Manner

**Figure 7:**
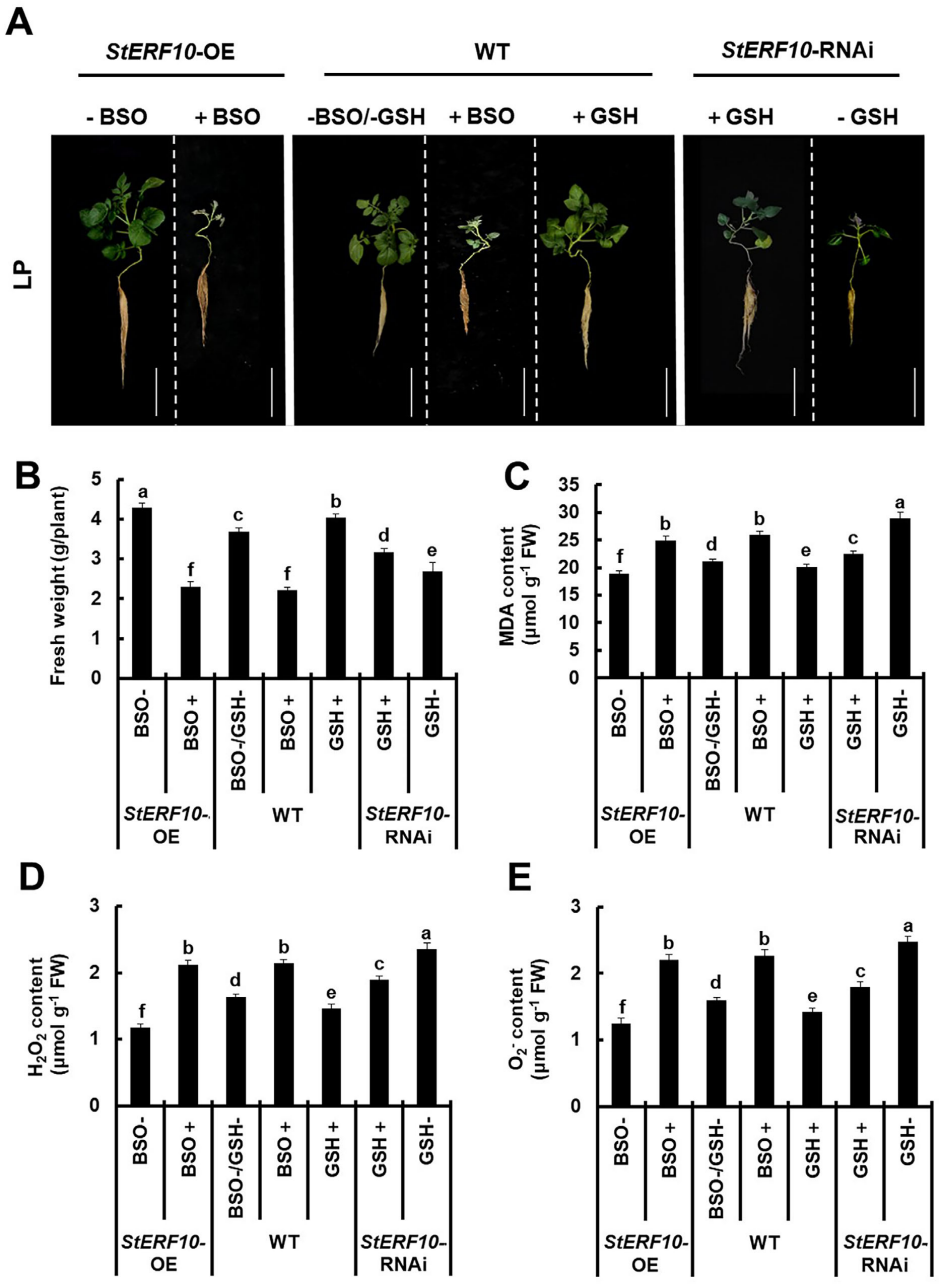
*StERF10* enhances LP stress adaptation in a GSH‐dependent manner in potato. (A) Phenotypes of *StERF10* transgenic potato under LP stress. *StERF10‐*OE and WT plants were sprayed with either water (‐BSO) or 0.1 mm BSO (+BSO); *StERF10‐*RNAi and WT plants were sprayed with either water (‐GSH) or 1 mm glutathione (+GSH). WT potato plants were treated with foliar applications of either 1.0 mm exogenous GSH or 0.1 mm L‐buthionine‐(S,R)‐sulfoximine (BSO), BSO is an inhibitor of GCL that suppresses GSH biosynthesis. Foliar sprays were applied every 3 days in the evening (20:00) to avoid GSH photolysis. Phenotypes were evaluated after all plants were grown in a LP hydroponic environment (0.1 mm Pi) for 20 days. Scale bar, 15 cm. (B) Fresh weight, (C) Malondialdehyde (MDA), (D) H_2_O_2_ and (E) O_2_^−^ content in shoots of wild type (WT), *StERF10*‐OE and RNAi plants shown in (A). The bars represent the mean value ± SD (*n* = 3 biological replicates, each replicate contained five individual plants). Statistical tests were performed by one‐way ANOVA, followed by Tukey's test. Different letters indicate significant difference (*p* < 0.05).

To investigate whether the enhancement of LP stress adaptation by *StERF10* depends on *StGSH1*‐mediated GSH accumulation, we applied BSO to inhibit GSH synthesis in LP‐stress‐treated *StERF10‐*OE and WT, and fed GSH to LP‐stress‐treated WT and *StERF10‐*RNAi lines. As observed previously, the *StERF10‐*OE lines performed better than the WT under LP stress (**Figure** **7**A). The growth of *StERF10‐*OE lines under LP stress was significantly reduced by BSO treatment and their biomass resembled that of the BSO‐treated WT (Figure 7B). As expected, *StERF10‐*OE lines displayed less accumulation of the membrane‐damage marker MDA, and the ROS (H_2_O_2_ and O_2_^−^), than the WT under LP stress (Figure 7C–E). The diminished accumulation of these stress‐associated metabolites in *StERF10‐*OE was abrogated by BSO‐induced inhibition of GSH synthesis. In contrast, LP‐stressed *StERF10‐*RNAi suffered more than the WT (Figure 7A,B) and accumulated higher levels of MDA, H_2_O_2_, and O_2_^−^ when compared to WT (Figure 7C–E). External application of GSH partially prevented the over‐accumulation of MDA, H_2_O_2_, and O_2_^−^ and significantly improved the growth of *StERF10‐*RNAi lines under LP stress. These results provide direct evidence that the StERF10‐triggered adaptation of potatoes toward LP stress is at least partially mediated by the activation of GSH synthesis, which is under the control of *StGSH1*.

## 3 Discussion

GSH, a central molecule of sulfur metabolism and redox homeostasis, modulates the antioxidant capacity of plants and is involved in stress responses.^[^ ^37^ ^]^ Although stress‐induced GSH accumulation has been well documented,^[^ ^3^, ^4^, ^8^, ^9^ ^]^ this study provides the first evidence demonstrating a causal relationship between GSH biosynthesis and plant adaptation to LP (Figure 1C–E). Notably, despite the abundance of phosphorus in the Earth's crust, the amount of effective Pi available for plant uptake is limited.^[^ ^38^ ^]^ Potato plants exhibit exceptionally high Pi demand during seedling establishment and tuber initiation, rendering adequate Pi acquisition critical for yield.^[^ ^25^, ^39^ ^]^ The application of exogenous GSH under LP stress led to a significant reduction in ROS levels (Figure 1G,H). Remarkably, exogenous GSH supplementation further attenuated LP‐induced ROS accumulation (Figure 1G,H), corroborating the antioxidant function of GSH in LP stress adaptation.

*StGSH1* encodes the rate‐limiting GCL that governs GSH biosynthesis and has been implicated in diverse abiotic stress responses.^[^ ^4^, ^12^ ^]^ However, its specific contribution to LP stress adaptation remains uncharacterized. In this study, we demonstrated that LP stress rapidly induces *StGSH1* expression (Figure 1F), initiating de novo GSH synthesis. Relative to WT plants, *StGSH1*‐overexpressing lines exhibited elevated GSH levels concomitant with the transcriptional activation of antioxidant enzymes (*StCAT1*, *StSOD2*, *StPOD66*, and *StAPX1*) that scavenge O_2_^−^ and H_2_O_2_ and reinforce the AsA‐GSH cycle (Figure 3B; Figure S7, Supporting Information). This coordinated redox buffering reduced lipid peroxidation, as evidenced by lower MDA accumulation compared to the wild‐type (Figure 3H), and liberated metabolic resources that supported biomass accumulation under LP stress (Figure 3F), implying that *StGSH1*‐mediated GSH synthesis not only mitigates oxidative damage but also optimizes metabolic processes to support plant growth.

During Pi starvation, plants rapidly remobilize internal Pi reserves by replacing phospholipids with non‐phosphorus glycolipids.^[^ ^36^, ^40^ ^]^ Key enzymes in this process include PLD, glycerophosphodiester phosphodiesterase (GDPD), and NPC.^[^ ^35^, ^36^ ^]^ We observed highly LP‐induced expression of phospholipid‐degrading genes, *StPLD* and *StNPC*, which was further enhanced in *StGSH1‐*overexpressing lines (Figure 4B). Consistent with this, PE content decreased in transgenic lines (Figure 4C), confirming enhanced phospholipid turnover and Pi reallocation.

Although research on the interplay between phosphorus and sulfur in plants remains limited, emerging evidence indicates that sulfur‐containing metabolites orchestrate Pi remobilization during phosphorus deficiency. In *Oryza sativa* and *Arabidopsis*, LP stress triggers substitution of phospholipids by sulfolipids to conserve Pi and sustain membrane integrity.^[^ ^41^, ^42^ ^]^ This sulfolipid synthesis compensates for phospholipid degradation to maintain membrane integrity. Sulfolipids such as SQDG can replace phospholipids, thereby preserving cellular function while conserving Pi.^[^ ^41^, ^42^, ^43^ ^]^ Here, LP stress triggered upregulation of sulfolipid synthase genes *StSQD1* and *StSQD2*, and this response was significantly amplified in *StGSH1*‐overexpressing potato lines (Figure 4B). Consequently, SQDG accumulation increased (Figure 4D), accompanied by elevated Pi release (Figure 4A). Conversely, *StGSH1*‐RNAi lines displayed impaired SQDG synthesis and Pi retention (Figure 4A,D). These results establish a direct mechanistic link between phosphorus remobilization and sulfur‐dependent GSH synthesis and Pi‐related lipid remodeling in potato. Unexpectedly, total P content did not differ among WT, *StGSH1*‐OE, and *StGSH1*‐RNAi lines (Figure S8A, Supporting Information), and transcript levels of phosphate transporters (*StPHT1;1* and *StPHO1*) remained unaltered among the genotypes (Figure S8B–D, Supporting Information). Collectively, these findings indicate that *StGSH1* likely contributes to potato adaptation to LP stress through mechanisms independent of modulating phosphorus content.

**Figure 8:**
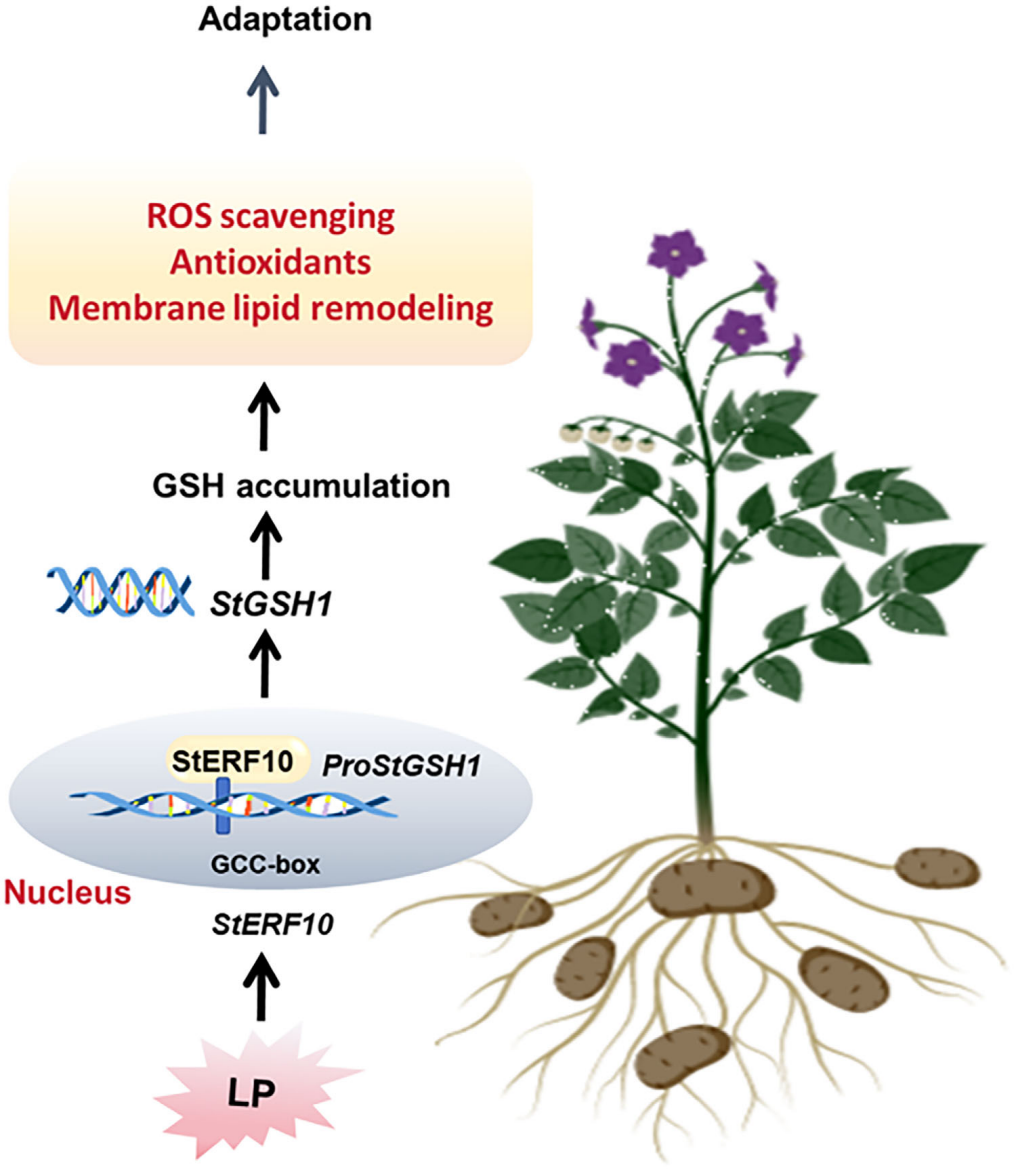
Working model of the StERF10‐StGSH1 module in response to LP stress in potato. The results demonstrate that LP stress induces the transcription factor *StERF10*, which directly activates the expression of *StGSH1*. StGSH1 catalyzes the rate‐limiting step of GSH biosynthesis. The enhanced GSH level prevents the over‐accumulation of deleterious ROS species, thereby protecting membrane integrity and, simultaneously, enables the provision of sulfite for UDP‐Sq synthesis, allowing efficient sulfolipid biosynthesis. The replacement of phospholipids with sulfolipids during membrane lipid remodeling subsequently liberates Pi from phospholipids, a significant sink of Pi in all eukaryotes.

To date, although existing studies have not elucidated whether ERF transcription factors directly modulate the transcription of genes associated with GSH biosynthesis, the role of ERF transcription factors in plant antioxidant defense has been extensively studied. For example, some ERF transcription factors were able to enhance the antioxidant capacity of plants by regulating the expression of antioxidant enzyme genes,^[^ ^44^ ^]^ and *Lycium barbarum LchERF* has been shown to enhance cadmium tolerance in transgenic tobacco, suggesting that the underlying mechanism may involve the modulation of GSH biosynthetic pathways.^[^ ^45^ ^]^ Critically, our work addresses this knowledge gap: we demonstrated that *StERF10* directly binds to the *StGSH1* promoter to activate transcription, thereby increasing glutamate‐cysteine ligase abundance and subsequently elevating cellular GSH pools to sustain potato growth under LP stress (Figures 5 and 6). The resulting GSH surge attenuated oxidative damage, as evidenced by the fact that exogenous GSH supplementation partially rescued the hypersensitive phenotype of *StERF10*‐RNAi lines under LP stress (Figure 7). Collectively, these findings uncover that *StERF10* plays a pivotal role in regulating the adaptive response to LP stress, through a linear transcriptional‐metabolic cascade: predominantly via the modulation of *StGSH1*‐mediated GSH biosynthesis, which subsequently enhances ROS scavenging and orchestrates membrane lipid remodeling, thereby optimizing Pi utilization during LP stress (**Figure** **8**). Although StERF10 is nuclear localized and StGSH1 is plastid‐targeted, StERF10 transcriptional activation of *StGSH1* happens in the nucleus. The newly synthesized StGSH1 protein is imported into plastids, where GSH synthesis occurs. These findings provide robust mechanistic insight into ERF‐mediated control of GSH‐dependent tolerance to oxidative stresses, such as LP, in potato.

In conclusion, this research establishes that LP stress induces the AP2/ERF transcription factor StERF10, which directly binds to the *StGSH1* promoter and activates glutamate‐cysteine ligase expression, leading to an increase of GSH pools (Figure 8). The resulting rise in GSH sustains cellular redox balance by participating in the AsA‐GSH cycle, and acts as a signaling metabolite that upregulates ROS scavenging genes, thereby reducing ROS accumulation and alleviating LP‐induced oxidative damage and growth inhibition (Figure 8). In addition, increased sulfur metabolic flux promotes membrane lipid remodeling and release of available phosphorus to maintain Pi homeostasis. The StERF10‐StGSH1 regulatory module provides a prime biotechnological target: its genetic or biochemical potentiation could markedly increase phosphorus‐use efficiency in potato, reduce fertilizer input, and sustain yield under Pi‐limiting conditions. GSH‐centered sulfur metabolism thus provides tractable targets for breeding LP‐tolerant cultivars. Future work should dissect the crosstalk between GSH, sulfur assimilation, and hormone signaling to fully understand the mechanistic basis of plant LP stress tolerance.

## 4 Experimental Section

### Plant Materials and Treatment

The potato genotype used in this study, *Solanum tuberosum* cv. E‐potato 3 (E3), was generously provided by Prof. Conghua Xie (Huazhong Agricultural University, China). Sterile seedlings were grown in controlled conditions (22 ± 2 °C, 2000 lx light intensity, 16‐h light/8‐h dark photoperiod). After 3 weeks in a standard Hoagland's nutrient solution, uniform plants were transferred to nutrient solutions with either 1.0 mm KH_2_PO_4_ (normal Pi; NP), or 0.1 mm KH_2_PO_4_ (low‐Pi; LP) and cultivated for an additional 10–20 days.^[^ ^46^ ^]^ A minimum of 15 plants in three biological replicates were used for phenotypic and biochemical analysis. For exogenous GSH or BSO treatment, 1 mm GSH or 0.1 mm BSO was used during the growth of potato plants. Electrolyte leakage (EL) was quantified using a conductivity meter (DDS‐307A, Hinotek) as described in a previous method.^[^ ^34^ ^]^ H_2_O_2_ and O_2_^−^ content was measured by titanium sulfate colorimetry and hydroxylamine method, respectively. CAT activity was assayed via H_2_O_2_ decomposition (240 nm); SOD via NBT photoreduction inhibition; POD by guaiacol oxidation (470 nm); APX via ascorbate oxidation rate (290 nm); MDA concentration using thiobarbituric acid reaction (532 nm). All assays were performed using commercial kits (Suzhou Comin Biotechnology Co., Ltd., Suzhou, China).^[^ ^47^ ^]^

### StGSH1 Protein Sequence Analysis

The StGSH1 coding sequence was obtained from the *Solanum tuberosum* genome v4.03 (https://phytozome‐next.jgi.doe.gov/info/Stuberosum_v4_03) by performing BLAST searches and comparative analysis in the Phytozome (http://www.phytozome.net) and NCBI (http://www.ncbi.nlm.nih.gov/) databases. Phylogenetic analysis was performed to infer the evolutionary relationships of StGSH1 using the maximum likelihood method in MEGA 7.0 (http://www.megasoftware.net/).^[^ ^48^ ^]^

### Vector Construction and Genetic Transformation

To obtain *StGSH1*‐overexpressing transgenic potatoes, the full‐length coding sequence (1572 bp) of *StGSH1* was inserted into the pTF101s vector under the control of a dual CaMV35S promoter. A 218‐bp *StGSH1‐*specific fragment was inserted into the RNAi vector pK7GWIWG2D(II) to generate *StGSH1*‐RNAi lines. Similarly, the 846‐bp full‐length CDS and a 225‐bp specific fragment of *StERF10* were amplified by PCR and ligated into the overexpression vector pCAMBIA2300 and the RNAi vector pK7GWIWG2D(II), respectively. All the recombinant plasmids were introduced into potato plants by *Agrobacterium*‐mediated methods as described previously.^[^ ^49^ ^]^ Primer sequences are listed in Table S2 (Supporting Information).

### StGSH1 Prokaryotic Expression and Purification

A 1350‐bp codon‐optimized fragment of *StGSH1* was cloned into the vector pET32a and then transformed into *Escherichia coli* BL21(DE3) competent cells. Recombinant protein expression was induced in cultures at 28 °C for 16 h to maximize solubility. The recombinant His_6_‐StGSH1 fusion was purified on Ni^2^⁺ resin, and the His tag was removed by TEV protease digestion to prevent potential interference from the His tag in subsequent enzyme activity assays. Enzyme activity was quantified spectrophotometrically at 340 nm by coupling ADP formation to the pyruvate kinase/lactate dehydrogenase system in Mg^2^⁺‐buffered reaction mixtures. To determine whether StGSH1 protein can form multimers, the target proteins were collected under the elution of 100–300 mm imidazole, concentrated to 10 mg mL^−1^, centrifuged at 12 000 × g for 10 min at 4 °C, and passed through a SD200 molecular sieve column. Subsequent separation of StGSH1 monomers and dimers was performed using Mono Q (anion exchange column), and the separated components were analyzed by SDS‐PAGE. Primers used for all constructs are listed in Table S2 (Supporting Information).

### Protein Crystallographic Characterization

Purified StGSH1 was concentrated to 1 mm in the purification buffer containing 25 mm Tris (pH 7.5). The crystallization condition was optimized and refined in 0.17 M ammonium acetate, 22% PEG3350 and 0.1 m Bis‐Tris (pH 5.5). Crystals were screened by automated sitting‐drop vapor diffusion method. The native StGSH1 data were collected at beamline BL17U1 of the Synchrotron Radiation Facility at Shanghai, China. All the data were processed and scaled by the HKL2000. The initial model was built using Phenix and subsequently manually rebuilt in Coot. The structure was finalized through refinement with Phenix. Detailed X­ray data collection and structure refinement statistics are shown in Table S1 (Supporting Information). The structure of StGSH1 was deposited in the Protein Data Bank (PDB) under accession number 9 UFC. To explore substrate recognition, protein‐substrate docking was performed using AutoDock (v4.2.6, http://autodock.scripps.edu) and the resulting complexes were visualized and analyzed in PyMOL (v2.5.4, https://pymol.org/2/).

### Measurement of StGSH1 Enzyme Activity

StGSH1 enzyme activity was analyzed by monitoring the rate of ADP production. This was measured spectrophotometrically at 340 nm (A_340_) using a coupled enzyme assay with pyruvate kinase and lactate dehydrogenase in an Mg^2+^‐buffered reaction system. The standard reaction system (0.5 mL) contained 100 mm Tris (pH 8.0), 150 mm KCl, 20 mm MgCl_2_, 10 mm L‐cysteine, 20 mm L‐glutamate, 5 mm ATP, 2 mm phosphoenolpyruvate, 0.2 mm NADH, 5 U of pyruvate kinase, and 10 U of lactate dehydrogenase. Initial velocity measurements were performed to determine the steady‐state kinetic parameters. The kinetic data were fitted to the Michaelis–Menten equation. The optimum temperature and pH for StGSH1 enzyme reaction were determined following the description above.

### Subcellular Localization

The full‐length CDS of *StGSH1* was cloned into the pCAMBIA2300‐GFP vector. For transient expression in protoplasts, potato leaf strips were enzymatically macerated for 3–4 h. The released protoplasts were isolated by sequential washing with W5 solution, including filtration through a 300‐mesh sieve and centrifugation at 100×g, and were subsequently resuspended in MMG solution. For transfection, the *CaMV35S::StGSH1::GFP* construct was introduced into 200 µL protoplasts using a PEG4000‐mediated method. For stable expression in plants, transgenic potato lines expressing *CaMV35S::StGSH1::GFP* were cultured for 2 weeks, and root tips from these plants were used for DAPI staining. All primers used are listed in Table S2 (Supporting Information).

### GUS Staining Analysis

A 1783‐bp fragment of the *StGSH1* promoter was cloned into the pTF102 vector to generate *proStGSH1*‐GUS transgenic potato plants. GUS staining performed as previously described.^[^ ^50^ ^]^ Briefly, 2‐week‐old *proStGSH1*‐GUS seedling were immersed in GUS staining solution and subjected to vacuum infiltration for 30 min. After overnight incubation at 37 °C, the seedling were decolorized using a graded ethanol series (70% to 90%). For transcriptional activation assays, *Nicotiana benthamiana* leaves were co‐infiltrated with *Agrobacterium* strains carrying the *StERF10*‐pCAMBIA2300 effector construct and the *proStGSH1*‐GUS reporter construct. After 48 h, GUS activity was quantified, and histochemical staining was imaged using the method described above. All primers used are listed in Table S2 (Supporting Information).

### Dual‐Luciferase Assays

The full‐length ORFs of the transcription factors *StMYB308*, *StHSFb.4*, and *StERF10* were individually cloned into the effector vector pGreenII62‐SK under the control of the *CaMV 35S* promoter. A 1783‐bp promoter fragment of *StGSH1* was cloned into the pGreenII0800‐LUC vector to generate the reporter construct. The recombinant effector and reporter constructs were then co‐expressed in *Nicotiana benthamiana* leaves by *Agrobacterium*‐mediated transient transformation. After 48 h, leaf samples were collected, and the activities of firefly luciferase (LUC) and *Renilla* luciferase (REN) were measured. Luminescence signals were detected using an Infinite M200 microplate reader (Tecan, Switzerland). All primers used are shown in Table S2 (Supporting Information).

### Yeast One‐Hybrid Assay

A yeast one‐hybrid cDNA library (OE Biotech, Shanghai) was generated from mRNA isolated from potato leaves, and screened against a bait yeast strain carrying the *StGSH1* promoter fused to a reporter gene in the pBait‐AbAi vector. Candidate transcription factors that potentially bind directly to the *StGSH1* promoter were identified from the primary screen. A credible candidate, *StERF10*, was further verified through pairwise retransformation assays. A 1783‐bp fragment of the *StGSH1* promoter, which contains a GCC‐box element, was cloned into the pAbAi vector and integrated into the YIH yeast genome to generate bait reporter. The minimum inhibitory concentration of aureomycin A (AbA) for this bait strain was determined on SD/‐Ura plates. The full‐length CDS of *StERF10* was cloned into the pGADT7 vector to generate the prey construct. The *StERF10‐*pGADT7 prey vector was then transformed into the bait yeast, and the resulting recombinant yeast cells were plated on the SD/‐Leu medium containing the selective concentration of AbA and incubated for 3 d. All primers used are listed in Table S2 (Supporting Information).

### Chromatin Immunoprecipitation Quantitative PCR (ChIP‐qPCR) Analysis

The recombinant plasmid *StERF10*‐GFP was introduced into *Agrobacterium tumefaciens* GV3101 and transiently expressed in potato leaves. Leaves infiltrated with an empty vector (GFP only) served as the control. Chromatin immunoprecipitation was performed using the EpiQucik Plant ChIP Kit (Epigentek, New York, USA). Cross‐linked chromatin samples were isolated and immunoprecipitated with anti‐GFP antibody. The eluted DNA fragments were used to amplify and quantify two specific regions of the *StGSH1* promoter: *Pro*StGSH1‐a (−1153 to −978 bp) and *Pro*StGSH1‐b (−447 to −236 bp). The amplification primers are listed in Table S2 (Supporting Information).

### Electrophoretic Mobility Shift Assay (EMSA)

The full‐length coding sequence of *StERF10* was cloned into the pET‐32a expression vector. The resulting StERF10‐His recombinant protein was induced and expressed in *Escherichia coli* BL21(DE3) cells and purified using a His‐tagged Protein Purification Kit (Beyotime). 5′‐Biotin‐labeled DNA probes were synthesized by Shanghai Biotechnology Co., Ltd. EMSA was then performed using the LightShift Chemiluminescent EMSA Kit (Thermo Fisher Scientific Inc., USA).

### Determination of Pi Content

The Pi concentration was measured using a modified malachite green colorimetric method.^[^ ^51^ ^]^ Briefly, 20 mg of the fresh sample was homogenized in 1 mL of 1% (v/v) glacial acetic acid. The homogenate was centrifuged at 10 000 × g for 15 min at 4 °C, and the supernatant was collected. The absorbance of the supernatant was then measured at 650 nm.

### Glutathione Extraction and Determination

Reduced (GSH) and oxidized glutathione (GSSG) were extracted and quantified using a modified protocol from previous reports.^[^ ^47^, ^52^ ^]^ Briefly, 100 mg of fresh potato leaves were ground in liquid nitrogen and homogenized in 0.5 mL of ice‐cold extraction buffer (50 mm phosphate buffer, pH 7.0, 1 mm EDTA). After homogenization, the extract was centrifuged at 12 000 × g for 15 min at 4 °C. A 100 µL aliquot of the supernatant was treated with pre‐cooled N‐ethylmaleimide (NEM, 1:10 v/v; Sigma–Aldrich #E3876) to derivatize free thiol groups, followed by deproteinization with 5% sulfosalicylic acid. GSH and GSSG were separated by HPLC (Agilent 1260, ZORBAX SB‐C18 column, 4 µm) under isocratic elution with 0.1% TFA in methanol (95:5, v/v) at a flow rate of 1.3 mL min^−1^ and a column temperature of 37 °C. Quantification was performed using standard curves generated from authentic GSH (Sigma #G4251) and GSSG (Sigma #G4376). The total glutathione (T‐GSH) content was calculated as GSH + 2×GSSG.

### Phospholipids and Sulfolipids Extraction and Determination

Lipid were extracted according to a previously described method with minor modifications.^[^ ^38^ ^]^ Briefly, 1 g of leaf was homogenized in isopropanol/MTBE containing 0.01% BHT and formic acid. The homogenate was then ultrasonicated on ice to disrupt chloroplasts and facilitate the release of sulfolipids such as sulfoquinovosyldiacylglycerol (SQDG). Lipids were subsequently extracted by sequential vortexing and ultrasonication. After drying under a nitrogen stream, the lipid extracts were reconstituted in acetonitrile/isopropanol/ammonium formate (65:30:5) for HPLC‐ELSD. Phosphatidylethanolamine (PE) and SQDG were separated and quantified using C18 column (with an acetonitrile‐isopropanol gradient) and HILIC column (with an acetonitrile‐water gradient), respectively. Lipid compounds were normalized with reference to Pant et al (2015).^[^ ^29^ ^]^

### RNA Extraction and RT‐qPCR Reactions

Total RNA was extracted using the RNAprep Plant Kit (Tiangen, Beijing, China), and then reverse‐transcribed into cDNA using PrimeScript RT kit and gDNA Eraser (TaKaRa Company, Dalian, China). Real‐time quantitative PCR (RT‐qPCR) was performed as described by Li et al. (2022).^[^ ^47^ ^]^ Primer specificity was confirmed by both sequence alignment using NCBI Primer‐BLAST and analysis of RT‐qPCR melting curves. Three biological replicates were used for each RT‐qPCR assay and normalization was performed using *StEF1α* (Accession Number: AB061263) as the reference gene, which was stably expressed in potato under various stress conditions.^[^ ^53^ ^]^ All the primers used are shown in Table S2 (Supporting Information).

### Statistical Analysis

All data are presented as the mean ± standard deviation (SD) of three independent biological replicates (*n* = 3). Statistical analyses were performed using SPSS software (version 24.0). Differences between two groups were assessed by Student's *t*‐tests, with statistical significance set at *p* < 0.05. Comparisons among more than two groups were performed using one‐way ANOVA, followed by a Tukey's post hoc test. Statistical significance was defined as a *p*‐value of less than 0.05 for all tests. Detailed statistical methods for each experiment are provided in the corresponding figure legends.

## Conflict of Interest

The authors declare no conflict of interest.

## Author Contributions

H.Z., Z.L., and S.L. conceived and supported this study. H.Z., M.L., and X.T. designed this research. X.H., X.Z., and X.T. performed experiments. X.T., X.H., S.F., and J.T. analyzed the data. X.T. and H.Z. wrote the manuscript. M.W. contributed to data interpretation and manuscript design. Z.L. and H.Z. supervised the study. All authors read and approved the final manuscript.

## Supporting information



Supporting Information

## Data Availability

The data that support the findings of this study are available in the supplementary material of this article.
