# Supplementary material for: A GLUTAMATE CYSTEINE LIGASE Gene StGSH1 Regulated by StERF10 Enhanced Glutathione Accumulation and Adaptation to Low Phosphorus Stress in Potato
Source: Adv Sci (Weinh). 2025 Nov 27;13(8):e09143. doi: 10.1002/advs.202509143 (PMC12884754; doi:10.1002/advs.202509143)
Supplement: Supplementary file 1 — Supporting Information [file ADVS-13-e09143-s001.docx]

**Supporting information**


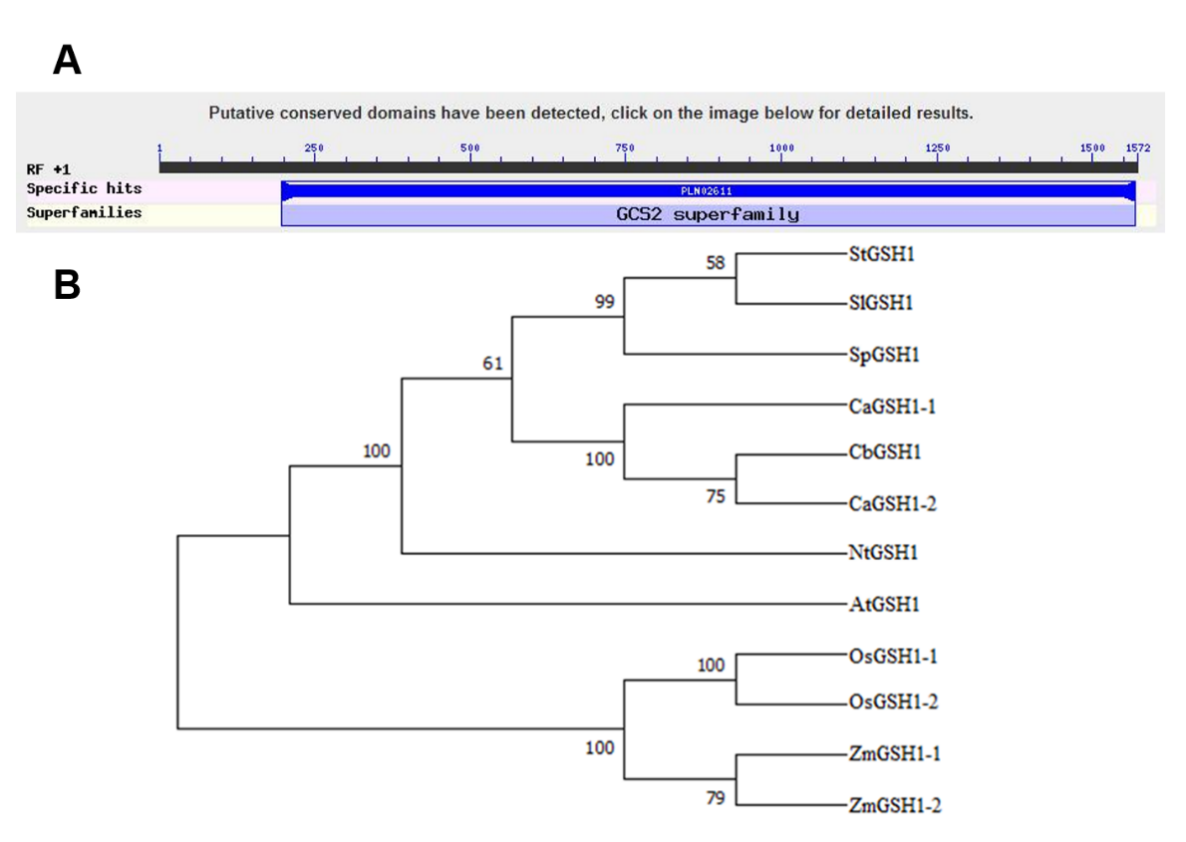


**Fig. S1** **Phylogenetic and conserved domain analysis of StGSH1. (A)** Predicted conserved domains within the StGSH1 protein sequence. **(B)** Phylogenetic tree of GSH1 proteins from various plant species, constructed using the maximum likelihood method. Species and accession numbers are as follows: *Solanum tuberosum* (StGSH1, XP006343987), *Solanum lycopersicum* (SlGSH1, ACQ91100), *Solanum pennellii* (SpGSH1, XP015085198), *Capsicum annuum* (CaGSH1-1, XP016566920; CaGSH1-2, PHT92417), *Capsicum baccatum* (CbGSH1, PHT57751), *Nicotiana tabacum* (NtGSH1, XP016460741), *Arabidopsis thaliana* (AtGSH1, NP194041), *Oryza sativa* (OsGSH1-1, CAD48599; OsGSH1-2, CAD48598), *Zea mays* (ZmGSH1-1, XP008648285; ZmGSH1-2, AQK95305).


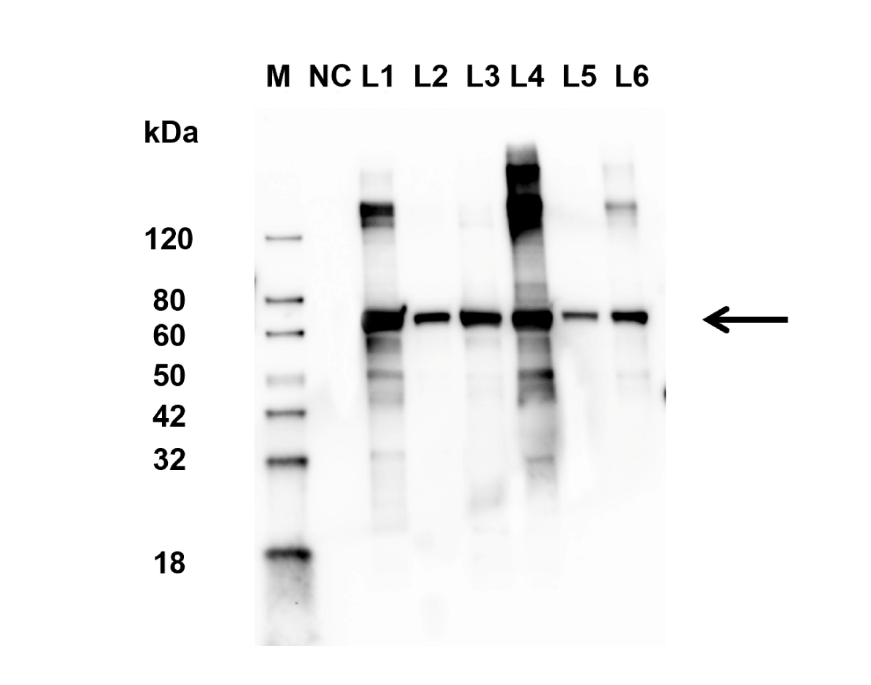


**Fig. S2** **Western blot analysis of recombinant StGSH1 protein expression in *Escherichia coli*.** M: Protein molecular weight marker (WB-MASTER, GenScript Biotech); NC: Non-induced cell lysate (negative control); L1: Whole-cell lysate after induction with 0.5 mM IPTG at 28℃ for 16 h; L2: Whole-cell lysate after induction with 0.5 mM IPTG at 20℃ for 4 h; L3: Soluble fraction (supernatant) from lysate induced at 28℃ for 16 h; L4: Insoluble fraction (pellet) from lysate induced at 28℃ for 16 h; L5: Insoluble fraction (pellet) from lysate induced at 20℃ for 4 h. L6: Debris of cell lysate with induction for 20℃ for 4 h. The pre-stained protein marker contains recombinant proteins with antibody-binding sites, allowing direct detection on blots.


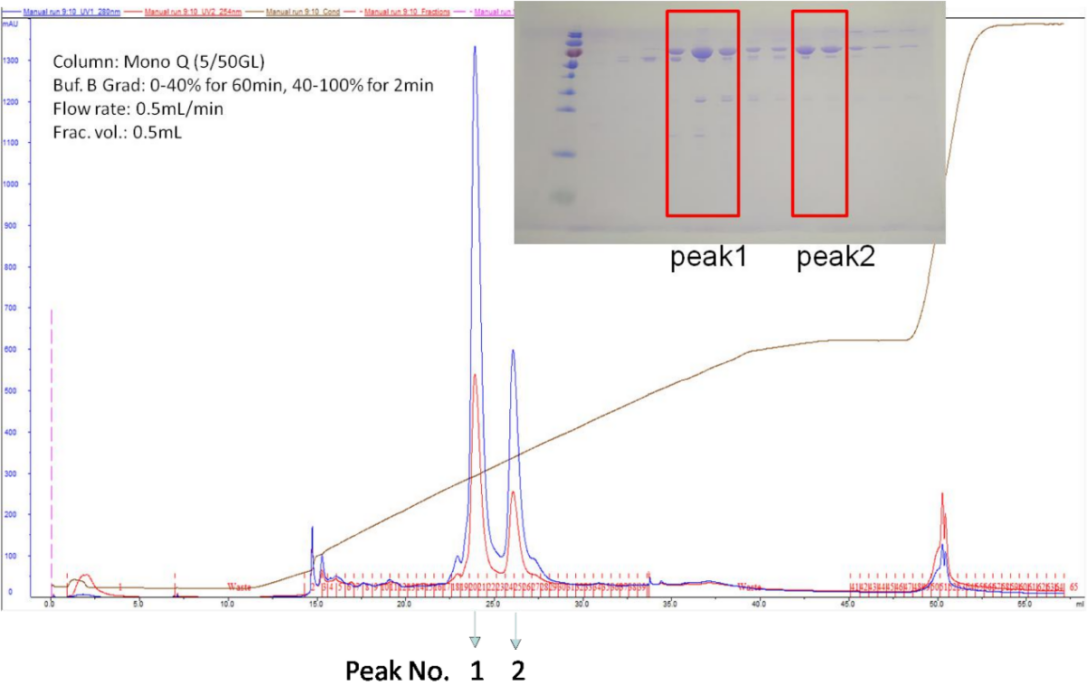


**Fig. S3** **Separation of StGSH1 dimers and monomers by anion-exchange chromatography.** Elution profiles showing the separation of two distinct protein populations. The collected fractions corresponding to Peak 1 and Peak 2 were analyzed by SDS-PAGE (inset), confirming their identities as the monomeric and dimeric forms of StGSH1, respectively.


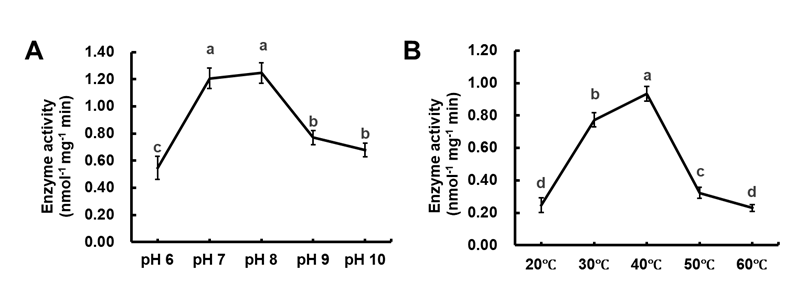


**Fig. S4 Biochemical characterization of StGSH1 enzyme activity.** **(A)** Optimum pH and **(B)** optimum temperature for the catalytic activity of the recombinant StGSH1 protein. Data are presented as mean value ± SD (n = 3 biological replicates). Statistical significance was determined by one-way ANOVA, followed by Tukey’s test. Different letters indicate significant difference at *P* < 0.05.

.
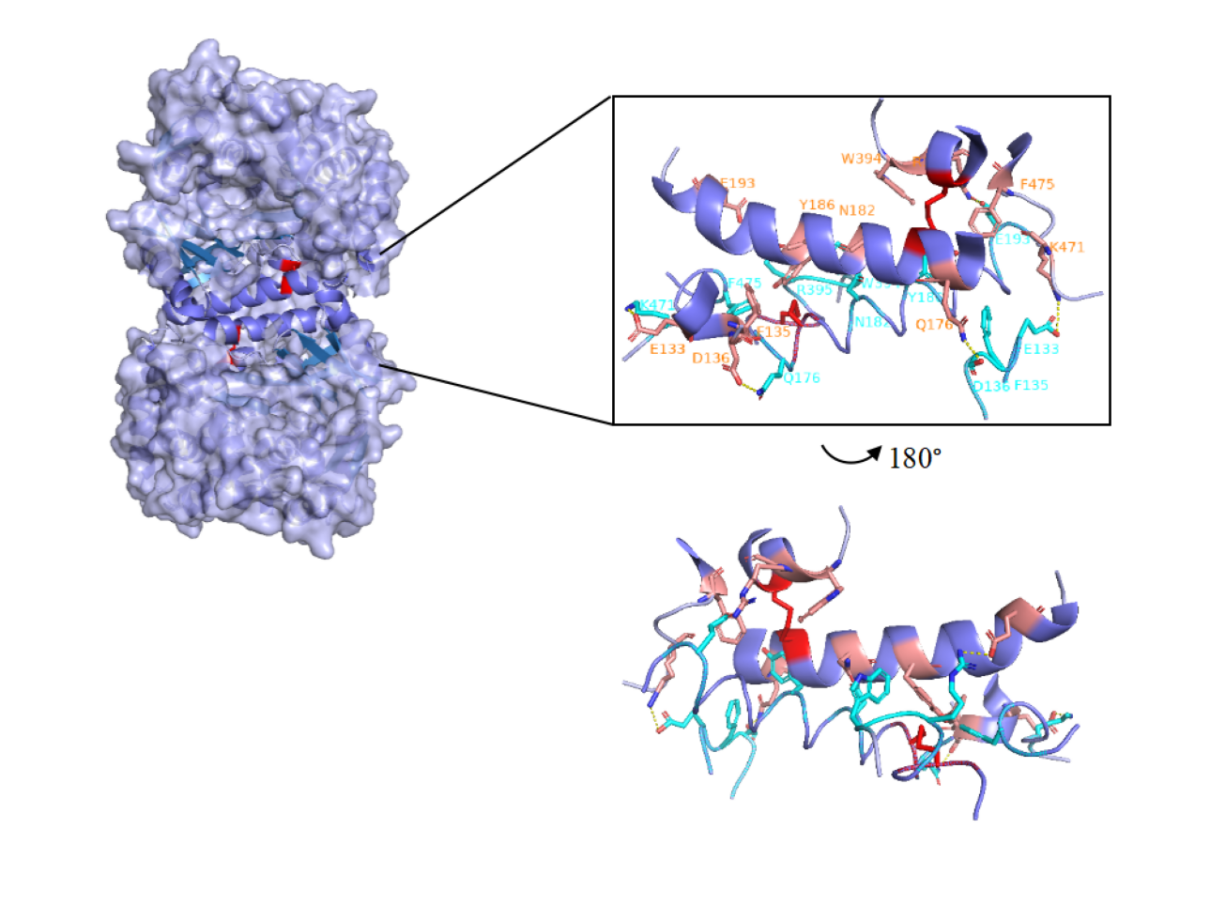


**Fig. S5** **Predicted dimerization interface of StGSH1.** The structural model suggests that dimer formation is stabilized by key residues at the subunit interface, including salt bridges (E133/R395, E193/K471, D136/Q176), aromatic stacking interactions (F135, W394, R395, F475), and polar contacts (N182, Y186).


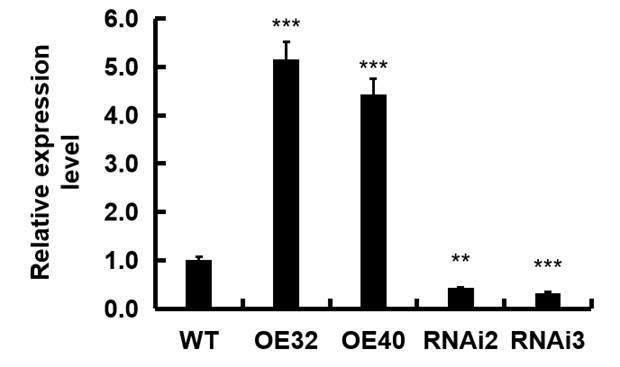


**Fig. S6** **Validation of *StGSH1* overexpression and silencing in transgenic potato lines.** Relative expression levels of *StGSH1* in leaves of wild-type (WT), *StGSH1-*OE (OE32 and OE40) and *StGSH1-*RNAi (RNAi2 and RNAi3) lines were determined by RT-qPCR. The bars represent the mean value ± SD (n = 3 biological replicates; each containing 3 individual plants). Student's *t*-test were used to determine statistical significance (**P* < 0.05, ***P* < 0.01, ****P* < 0.001).


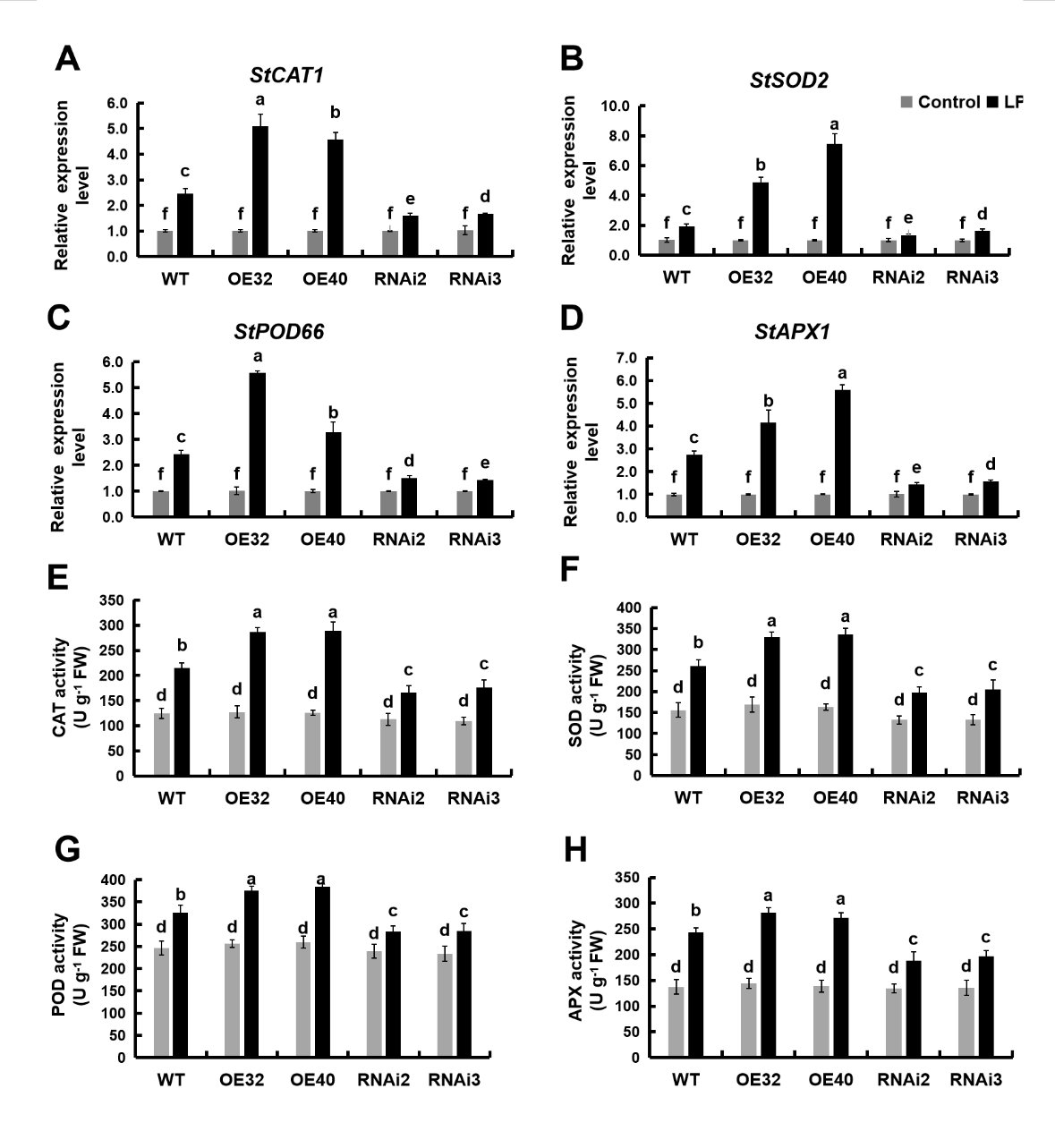


**Fig. S7** **The *StGSH1* enhances the transcriptional and enzymatic antioxidant capacity under LP stress.** Wild-type (WT), *StGSH1*-OE (OE32 and OE40), and *StGSH1*-RNAi (RNAi2 and RNAi3) plants were grown in Hoagland’s solution with normal phosphate (NP, 1.0 mM Pi) for 3 weeks and then subjected to NP or low phosphate (LP, 0.1 mM Pi) treatment for 15 days. **(A-D)** Relative transcript levels of the antioxidant genes **(A)** *StCAT1*, **(B)** *StSOD2*, **(C)** *StPOD66* and **(D)** *StAPX1* in leaves of the indicated genotypes under LP stress. **(E-H)** Corresponding activities of the antioxidant enzymes **(E)** catalase (CAT), **(F)** superoxide dismutase (SOD), **(G)** peroxidase (POD), and **(H)** ascorbate peroxidase (APX). Data are represented the mean value ± SD (n = 3 biological replicates). Statistical significance was determined by one-way ANOVA, followed by Tukey’s test. Different letters indicate significant difference at *P* < 0.05.


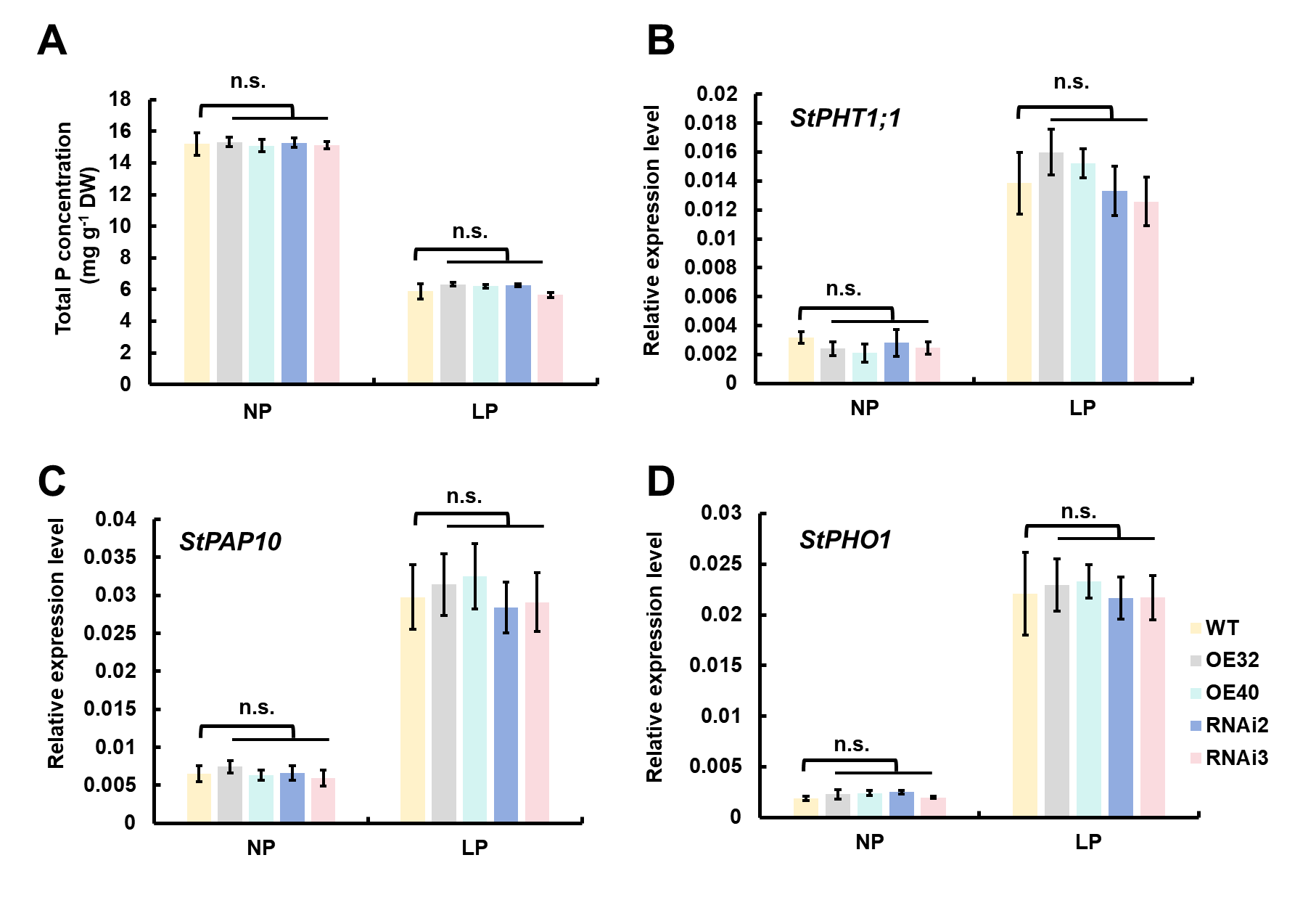


**Fig. S8 The *StGSH1* regulates LP adaptation without altering total phosphorus accumulation or the expression of phosphate starvation-induced (PSI) genes.** Wild-type (WT), *StGSH1*-OE (OE32, OE40), and *StGSH1*-RNAi (RNAi2 and RNAi3) plants were grown hydroponically for 3 weeks under normal phosphate (NP, 1.0 mM Pi) and then treated with NP or low phosphate (LP, 0.1 mM Pi) for 15 days. **(A)** Total phosphorus content in shoots of the plants described above. **(B-D)** Relative expression levels of the PSI genes *StPHT1;1* **(B)**, *StPHO1* **(C)**, and *StPAP10* **(D)** in root tips of WT and transgenic plants under LP stress described above. Data are presented as mean ± SD (n = 3 biological replicates). Student’s *t*-test was used to determine statistical significance (*p*<0.05); n.s., not significant.


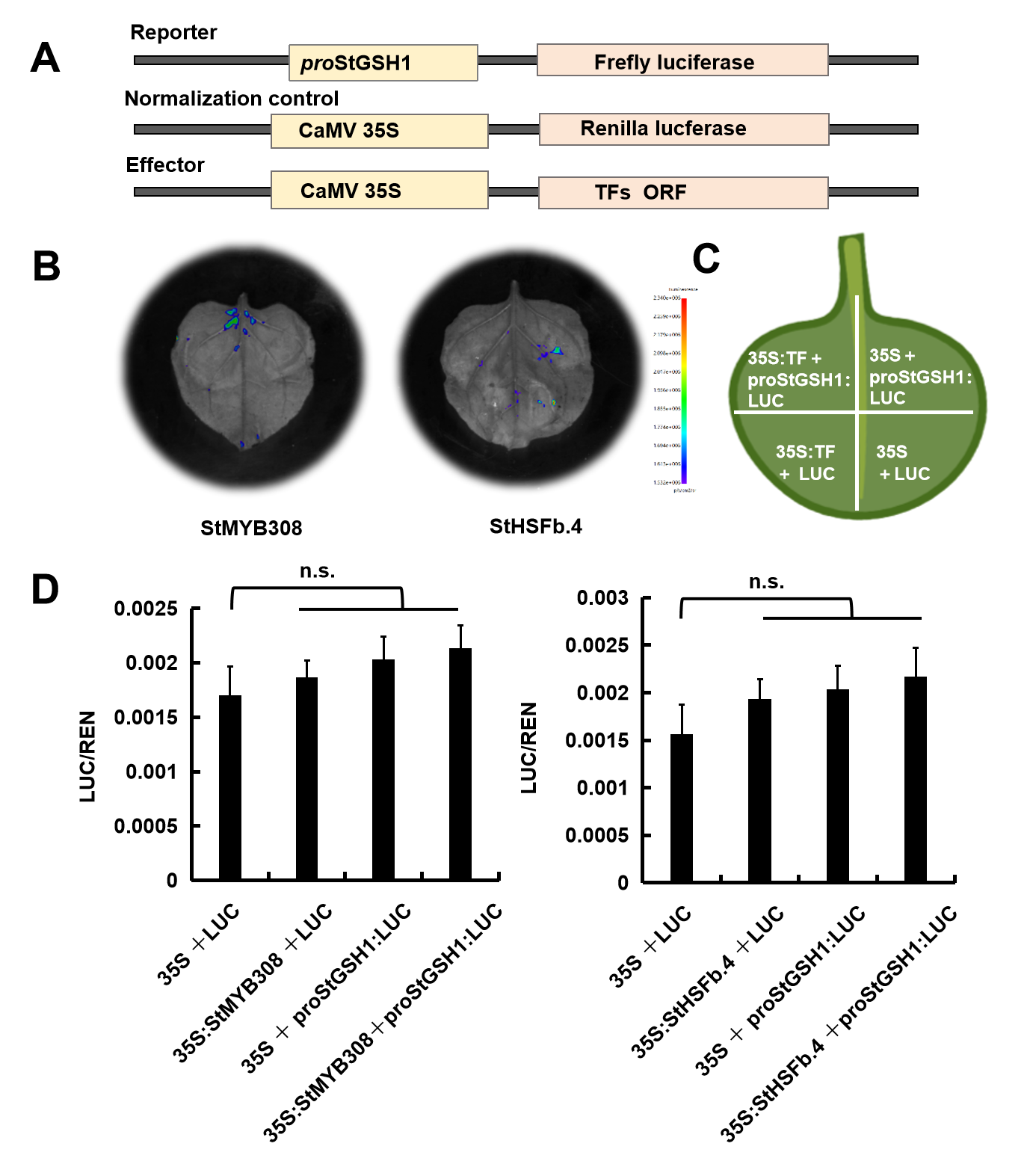


**Fig. S9** **Screening of LP-induced transcription factors for *StGSH1* transcriptional activation using a dual-luciferase (LUC) assay.** Two candidate transcription factors (TFs) StMYB308 and StHSFb.4 induced by LP stress were tested for their ability to bind to the *StGSH1* promoter. **(A)** Schematic of the effector and reporter constructs used in the assay. **(B)** Luminescence detection in *Nicotiana benthamiana* leaves following agroinfiltration with effector constructs (35S::StMYB308 or 35S::StHSFb.4) and the reporter construct (*proStGSH1*::LUC). **(C)** Detail of an *Agrobacterium*-infiltrated leaf area. **(D)** Quantification of LUC/REN ratios. The *proStGSH1*::LUC reporter or the empty pGreenII 0800-LUC vector was co-expressed with effectors driving TF expression (*35S::TF*) or the empty pGreenII 62-SK vector in *Nicotiana benthamiana* leaves. The bars represent the mean value ± SD (n = 3 biological replicates). Student’s *t*-test was used to determine statistical significance (*p*<0.05); n.s., not significant.


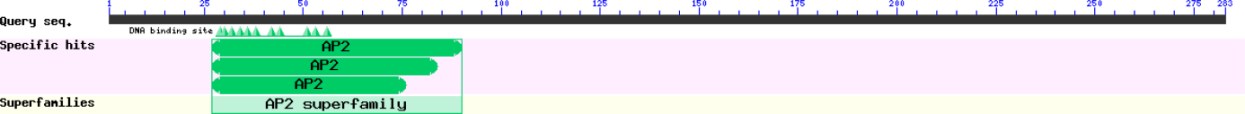


**Fig. S10 Conserved structural domains of the StERF10 protein.** The diagram illustrates the conserved AP2 DNA-binding domain identified in the StERF10 protein sequence.

**
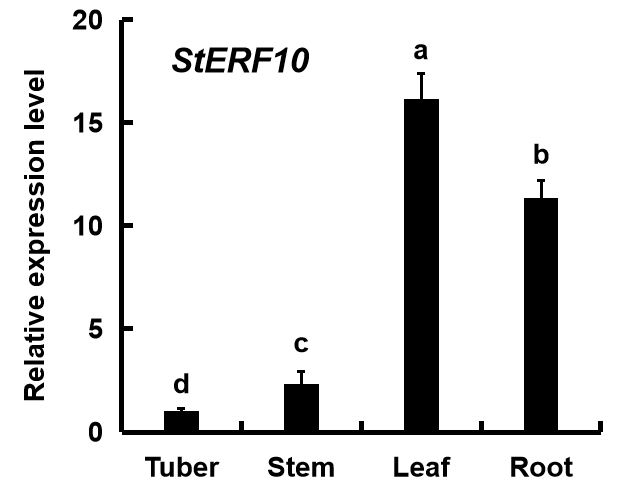
**

**Fig. S11** **Organ expression profile of *StERF10* in WT potato.** Relative transcript levels of *StERF10* in root, stem, leaf, and tuber of wild-type potato plants were determined by RT-qPCR. The transcript level of *StERF10* in tuber was set to 1.0. The bars represent the mean value ± SD (n = 3 biological replicates; each containing 3 individual plants). Statistical significance was determined by one-way ANOVA, followed by Tukey’s test. Different letters indicate significant difference at *P* < 0.05.


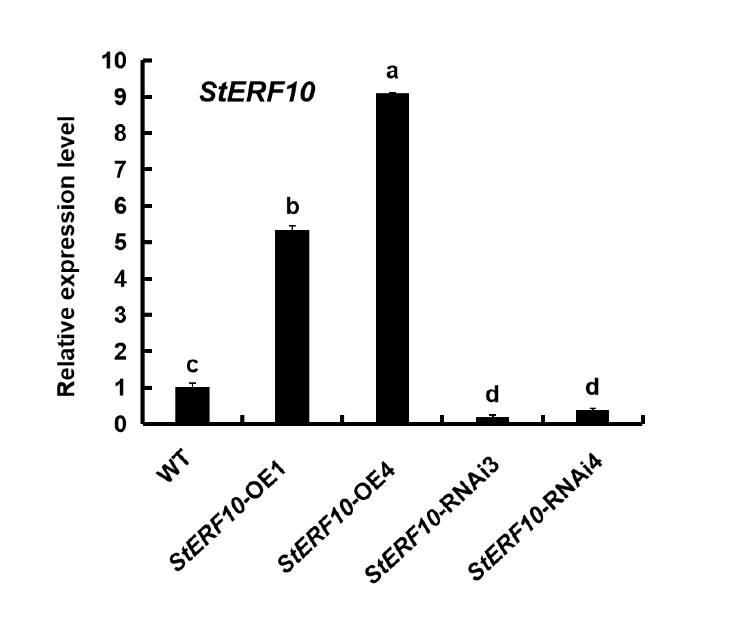


**Fig. S12** **The relative expression of *StERF10* in leaves of transgenic potato plants.** Expression levels were analyzed in wild-type (WT), *StERF10-*OE (OE1 and OE4) and *StERF10-*RNAi (RNAi3 and RNAi4) lines. The expression level in the WT was set to 1.0. The bars represent the mean value ± SD (n = 3 biological replicates; each containing 3 individual plants). Statistical significance was determined by one-way ANOVA, followed by Tukey’s test. Different letters indicate significant differences at *P* < 0.05.


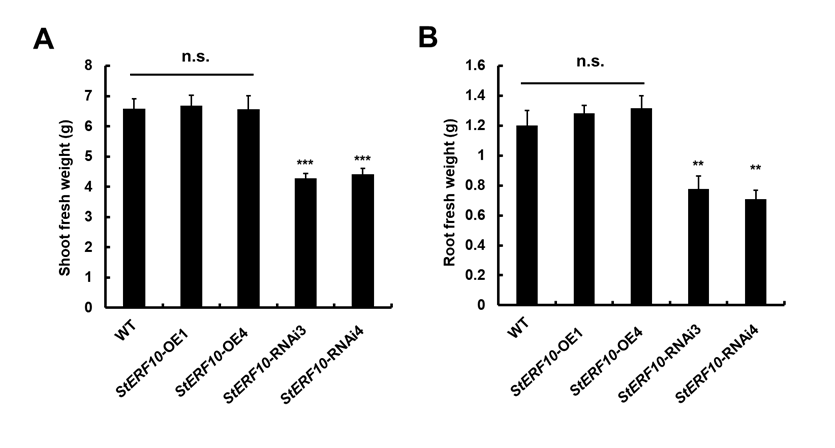


**Fig. S13 Fresh weight of hydroponically grown WT, *StERF10*-OE (OE1 and OE4), and *StERF10*-RNAi (RNAi3 and RNAi4) potato plants under normal phosphorus conditions. Four-week-old plants were grown in nutrient solution containing 1.0 mM KH₂PO₄ (NP). (A)** Shoot and **(B)** root fresh weight per plant. The bars represent the mean value ± SD (n = 3 biological replicates; each containing 5 individual plants). Student's *t*-test was used to determine statistical significance (***P* < 0.01, ****P* < 0.001); n.s., not significant.

**Supplementary Table S1**. Data collection and refinement statistic.

| **Data collection** |  |
| --- | --- |
| Data set | StGSH1 |
| PDB code | 9UFC |
| Wavelength (Å) | 0.979 |
| Resolution (Å) | 46.69 - 2.53 (2.62 - 2.53) |
| Space group | C222_1_ |
| Cell dimensions | 118.65 179.09 193.22 |
| (Å/degree) | 90 90 90 |
| Unique reflections | 67371 (6626) |
| Completeness (%) | 98.25 (97.42) |
| Rmeas (%) | 11.9 (100.8) |
| Rpim (%) | 5.0 (43.3) |
| Redundancy | 5.6(5.3) |
| Average *I*/σ(I) | 14.32 (1.2) |
| Wilson B value (Å^2^) | 48.02 |
|  |  |
| **Statistics for Refinement** |  |
| Resolution (Å) | 46.69 - 2.53 (2.62 - 2.53) |
| Rwork (%) | 19.12 (28.72) |
| Rfree (%) | 24.06 (31.90) |
| Reflections used | 67337 (6620) |
| R.m.s.d. |  |
| Bond (degree) | 0.65 |
| Length (Å) | 0.003 |
| No. of atoms | 14161 |
| Average B factors (Å^2^) | 59.56 |
| Ramachandran plot |  |
| Favored region (%) | 97.92 |
| Allowed region (%) | 2.08 |
| Outliers (%) | 0 |
| Note: values in parentheses are for the highest resolution shell. | |

**Supplementary Table S2.** Sequences of the PCR primers used in this study.

| **Primer Name** | **Sequence (5’-3’)** | **Primer annotation** |
| --- | --- | --- |
| StGSH1-CDS-F | ATGGCCTTGATGTCTCAGG | **Gene clone** |
| StGSH1-CDS-R | TCAATAGAGAAGCTCCTCAAAG |  |
| pTF101s-StGSH1-F | CCGGGGATCCTCTAGAATGGCCTTGATGTCTCAGG | **Overexpression** |
| pTF101s-StGSH1-R | GCAGGTCGACTCTAGATCAATAGAGAAGCTCCTCAA |  |
| 35S-2300-ERF10-KpnI-F | CGGGGGACGAGCTCGGTACCATGAAAACCACCATCAC | **GUS activity analysis; Overexpression** |
| 35S-2300-ERF10-XbaI-R | CACCATGGTGTCGACTCTAGAAGATTAAAATGGTACATAATT |  |
| StGSH1-detect-F | ATCCTCTAGAATGGCCTTG | **Identification** |
| StGSH1-detect-R | GACTCTAGATCAATAGAGAA |  |
| StGSH1-RNAi-F | CCGTAAGAAGAGGCAAGAGTATGAGGACTTTAGTTCTGAAGCTGAC | **RNAi** |
| StGSH1-RNAi-R | AATTCGCGGTACCCGGGGATCCTCTCCACATACTGCTCAAACCCAA |  |
| ERF10-RNAi-F | GGGGACAAGTTTGTACAAAAAAGCAGGCTCCATGAAAACCACCATCAC |  |
| ERF10-RNAi-R | GGGGACCACTTTGTACAAGAAAGCTGGGTCTCGTAGGGCTCGGGCAGC |  |
| pTF102-proStGSH1-F | CTATGACATGATTACGAATTCTGAGAATCTCGGTTCGAGAAATC | **GUS activity analysis** |
| pTF102-proStGSH1-R | GACTGACCTACCCGGGGATCCGCTTTGTGCCTGAATATCACAG |  |
| StGSH1-GFP-F | CGGGGGACGAGCTCGGATGGCCTTGATGTCTCAGG | **Subcellular localization** |
| StGSH1-GFP-R | CACCATGGTGTCGACTCTAGATAGAGAAGCTCCTCAAAGATAG |  |
| StERF10-GFP-F | CGGGGGACGAGCTCGGTACCATGAAAACCACCATCAC |  |
| StERF10-GFP-R | CACCATGGTGTCGACTCTAGAAGATTAAAATGGTACATAATT |  |
| pet32a-StGSH1-His-F | ATGGCTGATATCGGATCCGAATTCATGGCCTTGATGTCTCAGG | **Protein expression** |
| pet32a-StGSH1-His-R | GAGTGCGGCCGCAAGCTTGTCGACATAGAGAAGCTCCTCAAAG |  |
| qStGSH1-F | CAAGAGAAGCCACATTTG | **RT-qPCR** |
| qStGSH1-R | CCACATACTGCTCAAACCCA |  |
| qStEF1α -F | ATTGGAAACGGATATGCT |  |
| qStEF1α -R | TCCTTACCTGAACGCCTGTC |  |
| qStPLD-F | CATTTCCGTCTGGGTCAC |  |
| qStPLD-R | TCGTCAGCACAGGAAACCTC |  |
| qStGDPD-F | GAAGGAGCAGACCGCCT |  |
| qStGDPD-R | ATGCCTTGTAGACCGCCTTC |  |
| qStSQD1-F | TGATGCTTGCTTGCCCAG |  |
| qStSQD1-R | TTCTTAAACCTGGCGATGCT |  |
| qStSQD2-F | ACAACGCAGCCATTTGG |  |
| qStSQD2-R | TGGAGCCTACGCTTGAACAG |  |
| qHSFb.4-F | GAGTTCATCCTGAATATTC |  |
| qHSFb.4-R | AGATGAAGAAGGAGGCATG |  |
| qMYB308-F | GCAGTTGCGGTAGTGAAAG |  |
| qMYB308-R | CTTGGTTTCCAAAGTTCTATAG |  |
| qNAC100-F | ATGGAAGAAGTGAAAGAAG |  |
| qNAC100-R | CAGCAATAGCTCTTCCAG |  |
| qERF10-F | GAAGGATCATCAAGAAG |  |
| qERF10-R | ATTAAAATGGTACATAATT |  |
| qWRKY5-F | GAGAGTTGCCGGAATG |  |
| qWRKY5-R | GCCTGTAGTACCACACCAAG |  |
| qMYB102-F | ATGGGAAGGTCTCCTTG |  |
| qMYB102-R | AGAAATGTCATGCATGAAG |  |
| qABI5.2-F | CAAGGTTTCACGCCTCG |  |
| qABI5.2-R | GACGGGCGCGGAGCTTG |  |
| qMYB86-F | CCACGAATCGCGGAGAATG |  |
| qMYB86-R | ACAATTCCATGCAAGCTGATCT |  |
| qWRKY12-F | GATTGTCGTATGGTAATAAC |  |
| qWRKY12-R | GAAAGAGGTGAAACAATCAT |  |
| qCAT1-F | ACAACAAATGCTGGTGGTCCT |  |
| qCAT1-R | TCTTTTCGCGATCAAAAGTCG |  |
| qSOD2-F | TGCTGGTAAGGAGCATGGTG |  |
| qSOD2-R | GAGAGGAATCTGCTGGTCGG |  |
| qPOD66-F | GATGGTCCGCCTAACCTCTC |  |
| qPOD66-R | ATTGGCCCTTGACACCCTTC |  |
| qAPX1-F | AGGAACTCCTGAGTGGAGAGA |  |
| qAPX1-R | CAGCATCTGCAAATCCGAGC |  |
| HSFb.4-62sk-F | AGGAATTCGATATCAAGCTTATGGCTTTAATGCTAGAT | **LUC** |
| HSFb.4-62sk-R | GTACCGGGCCCCCCCTCGAGAGATGAAGAAGGAGGCATG |  |
| MYB308-62sk-F | AGGAATTCGATATCAAGCTTATGTTTTTGTTTCCTTATG |  |
| MYB308-62sk-R | GTACCGGGCCCCCCCTCGAGCTTGGTTTCCAAAGTTCTATAG |  |
| ERF10-62sk-F | AGGAATTCGATATCAAGCTTATGAAAACCACCATCAC |  |
| ERF10-62sk-R | GTACCGGGCCCCCCCTCGAGATTAAAATGGTACATAATT |  |
| 0800LUC-proGSH1-F | CGACGGTATCGATAAGCTTAGAATCTCGGTTCGAGAAATC |  |
| 0800LUC-proGSH1-R | CAGGAATTCGATATCAAGCTTGCTTTGTGCCTGAATATCACAG |  |
| ADT7-ERF10-EcoRI-F | CCATGGAGGCCAGTGAATTCATGAAAACCACCATCAC | **Y1H assay** |
| ADT7-ERF10-EcoRI-R | TGCCCACCCGGGTGGAATTCAGATTAAAATGGTACATAATT |  |
| pAbAi-proGSH1-KpnI-F | TGAATTCGAGCTCGGTACCAATCTCGGTTCGAGAAATC |  |
| pAbAi-proGSH1-XhoI-R | ACAGAGCACATGCCTCGAGTTGTGCCTGAATATCACAG |  |
| proGSH1-a-F | CTCTCTTTATAGCTTAACAAA | **Chip-qPCR** |
| proGSH1-a-R | AAAAGAAAAACTTTGTAGGGTT |  |
| proGSH1-b-F | GCACGATAGCAATATCAAGCCAC |  |
| proGSH1-b-R | CCAGAAGGCTCAATCACCAATCC |  |

**Supplementary Table S3.** Screening of candidate transcription factors that potentially bind directly to the *StGSH1* promoter using a yeast-one-hybrid cDNA library.

| **Gene ID** | **Name** | **Description** |
| --- | --- | --- |
| PGSC0003DMG400007962 | HSFb.4 | HSF domain class transcription factor |
| PGSC0003DMG400013215 | MYB308 | Transcription factor |
| PGSC0003DMG400014845 | NAC100 | Transcription factor |
| PGSC0003DMG400017051 | ERF10 | AP2/ERF domain-containing transcription factor |
| PGSC0003DMG400028469 | WRKY5 | WRKY transcription factor 5 |
| PGSC0003DMG400007325 | MYB86 | Transcription factor Myb |
| PGSC0003DMG400031175 | WRKY12 | Transcription factor |
| PGSC0003DMG400006408 | MYB102 | Transcription factor MYB102 |
| PGSC0003DMG400028121 | ABI5.2 | BZIP domain class transcription factor |
